# Kisspeptin-10 binding to Gpr54 in osteoclasts prevents bone loss by activating Dusp18-mediated dephosphorylation of Src

**DOI:** 10.1038/s41467-024-44852-9

**Published:** 2024-02-12

**Authors:** Zhenxi Li, Xinghai Yang, Ruifeng Fu, Zhipeng Wu, Shengzhao Xu, Jian Jiao, Ming Qian, Long Zhang, Chunbiao Wu, Tianying Xie, Jiqiang Yao, Zhixiang Wu, Wenjun Li, Guoli Ma, Yu You, Yihua Chen, Han-kun Zhang, Yiyun Cheng, Xiaolong Tang, Pengfei Wu, Gewei Lian, Haifeng Wei, Jian Zhao, Jianrong Xu, Lianzhong Ai, Stefan Siwko, Yue Wang, Jin Ding, Gaojie Song, Jian Luo, Mingyao Liu, Jianru Xiao

**Affiliations:** 1 Institute of Orthopedic Biomedical and Device Innovation, School of Health Science and Engineering, University of Shanghai for Science and Technology, Shanghai, 200093 China; 2 Institute of Orthopedics, Department of Orthopedic Oncology, Shanghai Changzheng Hospital, Naval Medical University, Shanghai, 200003 China; 3 East China Normal University and Shanghai Changzheng Hospital Joint Research Center for Orthopedic Oncology, Shanghai Key Laboratory of Regulatory Biology, Institute of Biomedical Sciences and School of Life Sciences, East China Normal University, Shanghai, 200241 China; 4 Department of Pathology, Beth Israel Deaconess Medical Center, Harvard Medical School, Boston, MA 02215 USA; 5 School of Biomedical Sciences, Hunan University, Changsha, 410082 China; 6 Department of Neurology, Beth Israel Deaconess Medical Center, Harvard Medical School, Boston, MA 02215 USA; 7 Academy of Integrative Medicine, Shanghai University of Traditional Chinese Medicine, Shanghai, 201203 China; 8 Department of Translational Medical Sciences, Institute of Biosciences and Technology, Texas A&M University Health Science Center, Houston, TX USA; 9 Shanghai Key Lab of Cell Engineering; Translational Medicine Research Center, Naval Medical University, Shanghai, 200433 China; 10 Clinical Cancer Institute, Center for Translational Medicine, Naval Medical University, Shanghai, 200433 China; 11 Yangzhi Rehabilitation Hospital (Shanghai Sunshine Rehabilitation Center), Tongji University School of Medicine, Shanghai, China

**Keywords:** Hormone receptors, Osteoporosis

## Abstract

Osteoclasts are over-activated as we age, which results in bone loss. *Src* deficiency in mice leads to severe osteopetrosis due to a functional defect in osteoclasts, indicating that Src function is essential in osteoclasts. G-protein-coupled receptors (GPCRs) are the targets for ∼35% of approved drugs but it is still unclear how GPCRs regulate Src kinase activity. Here, we reveal that GPR54 activation by its natural ligand Kisspeptin-10 (Kp-10) causes Dusp18 to dephosphorylate Src at Tyr 416. Mechanistically, Gpr54 recruits both active Src and the Dusp18 phosphatase at its proline/arginine-rich motif in its C terminus. We show that Kp-10 binding to Gpr54 leads to the up-regulation of Dusp18. *Kiss1*, *Gpr54* and *Dusp18* knockout mice all exhibit osteoclast hyperactivation and bone loss, and Kp-10 abrogated bone loss by suppressing osteoclast activity in vivo. Therefore, Kp-10/Gpr54 is a promising therapeutic target to abrogate bone resorption by Dusp18-mediated Src dephosphorylation.

## Abstract (web summary)

The authors of this manuscript identify that Gpr54 activation by its natural ligand Kisspeptin-10 can abrogate bone resorption. Mechanistically, Gpr54 recruits both active Src and the Dusp18 phosphatase, which causes Dusp18 to dephosphorylate Src at Tyr 416.

## Introduction

As we age, bone metabolism and homeostasis shift to favor over-activated osteoclasts, which leads to bone loss, a hallmark of human diseases such as osteoporosis^1,2^. Osteoclasts, which have the only capacity to resorb bone, are formed from bone marrow monocytes induced by macrophage colony-stimulating factor (M-CSF) and the receptor activator of nuclear factor-κB ligand (RANKL)^3,4^. Mechanically, both M-CSF and RANKL promote actin remodeling and bone resorption of osteoclasts, which mostly depends upon induction of Src kinase activation (phosphorylation at Y416)^5,6^. Src consists of 4 functional regions: Src homology 4 domain (SH4), SH3, SH2, SH1 (catalytic domain) and is activated through auto-phosphorylation at tyrosine 416 upon SH3 ligand binding^7–9^. Two main SH3 domain-binding motifs, proline/arginine-rich motifs (PR motifs), have been identified: R/KxxPxxP (class I) and PxxPxR/K (class II) (where K is lysine and x is any amino acid)^10–13^. Even though Src is normally present in a broad variety of cell types, genetic knockout of the *Src* gene in mice leads to only one major phenotype - severe osteopetrosis due to impaired osteoclast function^5,14–17^. Therefore, inhibition of Src kinase activity has been considered as a useful therapeutic strategy for osteoclast overactivation-mediated bone loss^18–22^.

G protein-coupled receptors (GPCRs) are the most important drug targets. It is estimated that ∼35% of marketed drugs act directly on GPCRs^23,24^. Src is activated by GPCRs via different ways including direct binding with Src through the SH3 binding motif in the intracellular domain^25–28^, indirect phosphorylation of Src at Y416 by Gα_s/i_^29^ or recruitment of Src via arrestins^30^. However, it is still entirely unclear how GPCRs adversely affect Src linase. GPR54, also named as KiSS1R (KiSS1 receptor), is a member of the GPCR superfamily^31^. Its natural ligands are Kisspeptins including Kp-54, −14, −13, and −10 encoded by the *KiSS1* gene^32^. Biologically, Kisspeptins/GPR54 signaling in hypothalamic neurons is the gatekeeper of puberty which regulates hormone release via the hypothalamic-pituitary-gonadal axis^33,34^. Kisspeptins/GPR54 triggers signaling cascades including Gq/11-PLCβ (phospholipase C β)-PKC (protein kinase C)/Ca^2+^ signal pathways, then the phosphorylation of MAP kinases, such as ERK1/2 and p38 are enhanced by PKC. In addition, KiSS1R activation recruits arrestin-1 and −2, which decrease and increase phosphorylated ERK1/2 levels respectively^35,36^. There is a PR motif in the C terminus of human GPR54 (GPR54 CT)^37^, which is conserved in terrestrial animals.

Here, we show Gpr54 recruits active Src and the phosphatase Dusp18 through the conserved PR motif upon activation by the ligand Kp-10. Furthermore, Kp-10/Gpr54 dose-dependently upregulates the expression of Dusp18, and Src (Y416) is dephosphorylated via Dusp18 upon Gpr54 activation by Kp-10, indicating that osteoclast activities are negatively regulated by Kp-10/Gpr54 at least partially through the dephosphorylation of Src (Y416) by Dusp18. In vivo, both whole-body (*Kiss1*^*−/−*^, *Gpr54*^*−/−*^*, Dusp18*^*−/−*^) and osteoclast conditional knockout (*Kiss1* cKO, *Gpr54* cKO) mice exhibit bone loss and osteoclast hyperactivation. Finally, we develop a bone-targeting Kp-10 consisting of six repetitive sequences of aspartate and serine^38^, (DSS)*6-Kp-10, that protects against bone loss in an ovariectomy (OVX)-induced osteoporosis model through blocking osteoclast activity. These results above suggest that Kp-10/Gpr54 plays a bone protective role as a negative osteoclast modulator during bone metabolism and may be a potent therapeutic target for the treatment of osteoclast-associated bone loss.

## Results

### Kp-10/Gpr54 govern osteoclast formation and bone resorption mainly by suppression of Src phosphorylation

**Fig. 1:**
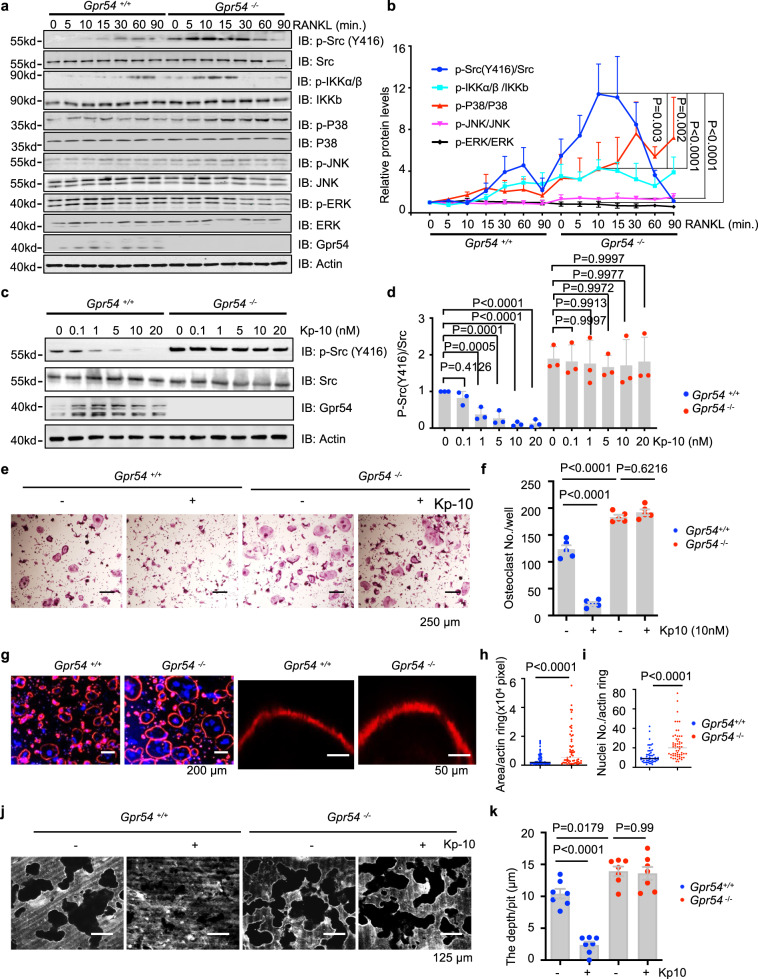
Kp-10 /Gpr54 governs osteoclasts formation and bone resorption mainly via suppression of Src phosphorylation. **a**–**d** IB analysis of whole cell lysates (WCL) derived from BMMs isolated from eight-week-old wild-type (WT) and *Gpr54*^*−/−*^ mice. BMMs were starved in serum-free α-MEM for 4 h and then treated with 100 ng/mL RANKL for different time (**a**) and quantification of protein levels (**b**), incubated with indicated dose of Kp-10 for 1 h (**c**) and quantification of protein levels (**d**). **e**–**i** BMMs isolated from eight-week-old WT and *Gpr54*^−/−^ mice were stimulated with M-CSF (10 ng/ml) and RANKL (50 ng/ml) for 5–7 days. Representative TRAP staining images are shown. Scale bar, 250 µm (**e**) and the number of osteoclasts/well was counted (**f**). Phalloidin staining of F-actin ring is shown. Scale bar, 250 µm, 50 µm (**g**), the area (**h**) and nuclei numbers (**i**) per actin ring were counted. **j**, **k** BMMs isolated from eight-week-old WT and *Gpr54*^−/−^ mice were cultured in vitro on dentin slices, and after incubation with M-CSF (10 ng/ml) and RANKL (50 ng/ml) for 5–7 days, the pits formed by osteoclast absorption activity were scanned by confocal microscopy (x, y, and z sections). Scale bar, 125 µm (**j**), and pits depth were quantified by confocal microscopy (**k**). Data represent means ± SEM. *P* values were determined by two-way ANOVA analysis (**b**), one-way ANOVA analysis (**d**, **f**, **k**), or two-tailed Student’s *t*-test (**h**, **i**), Representative results were obtained from at least three independent experiments. Source data are provided as a Source Data file.

The critical role of Src in osteoclast-mediated bone resorption has been shown by Src gene deletion in mice^14^. To examine the role of GPR54 in osteoclast, we detected changes in RANKL signal pathways when *Gpr54* was deleted. In contrast to MAPKs and NF-κB signaling pathways, Gpr54-deletion lead to an obvious change in Src (Y416) phosphorylation in bone marrow monocytes (BMMs) (Fig. 1a, b). Consistently, treatment of Kp-10 effectively reduced the phosphorylation of Src (Y416) in *Gpr54* WT osteoclasts, but failed to inhibit Src (Y416) phosphorylation in *Gpr54*^−/−^ osteoclasts (Fig. 1c, d). Furthermore, Src phosphorylation induced both by RANKL and M-CSF was suppressed after treatment with Kp-10 in WT osteoclasts, but not in *Gpr54*^−/−^ osteoclasts (Supplementary Fig. 1a, b). Additionally, levels of bone-derived Kisspeptins changed more significantly during osteoclast differentiation than in osteoblasts (Supplementary Fig. 1c). However, as the gatekeeper of puberty, Kisspeptins in serum declined in ovariectomized mice (considered as an age-related menopausal bone loss model) (Supplementary Fig. 1d).

Consistent with these findings, *Gpr54*-deletion enhanced osteoclast formation by TRAP staining (Fig. 1e) and the mean number of osteoclasts per well (Fig. 1f). Actin ring formation was also enhanced by *Gpr54* deletion (Fig. 1g–i). Additionally, bone resorption was promoted when *Gpr54* was deleted, which could be rescued upon ectopic expression of Gpr54 (Supplementary Fig. 2a, b). Furthermore, Kp-10 suppressed bone resorption in *Gpr54*-WT osteoclasts but did not have a significant effect in *Gpr54*^−/−^ osteoclasts (Fig. 1j, k). Remarkably, Gpr54 activation by Kp-10 also suppressed osteoclast formation in human giant cell tumor of bone (GCT) cells (Supplementary Fig. 2c, d). Consistently, Inhibition of Gpr54 by the GPR54 antagonist of GPR54 WB599 (2-acylamino-4,6-diphenylpyridines)^39^ enhanced osteoclast formation (Supplementary Fig. 2e, f). In keeping with these results, *Kiss1* loss also enhanced osteoclast formation as measured by the TRAP staining assay (Supplementary Fig. 2g) and the mean number of osteoclasts (Supplementary Fig. 2h). Furthermore, osteoclast resorption was promoted by *Kiss1* deletion, which was effectively rescued by ectopic expression of Kiss1 (Supplementary Fig. 2i, j). Taken together, these results indicated that Kp-10/Gpr54 played a negative role in osteoclast formation and bone resorption mainly via inhibition of Src phosphorylation.

### GPR54 activation by the ligand Kp-10 recruits the Src kinase via the PR motif in the GPR54 C-Terminus

**Fig. 2:**
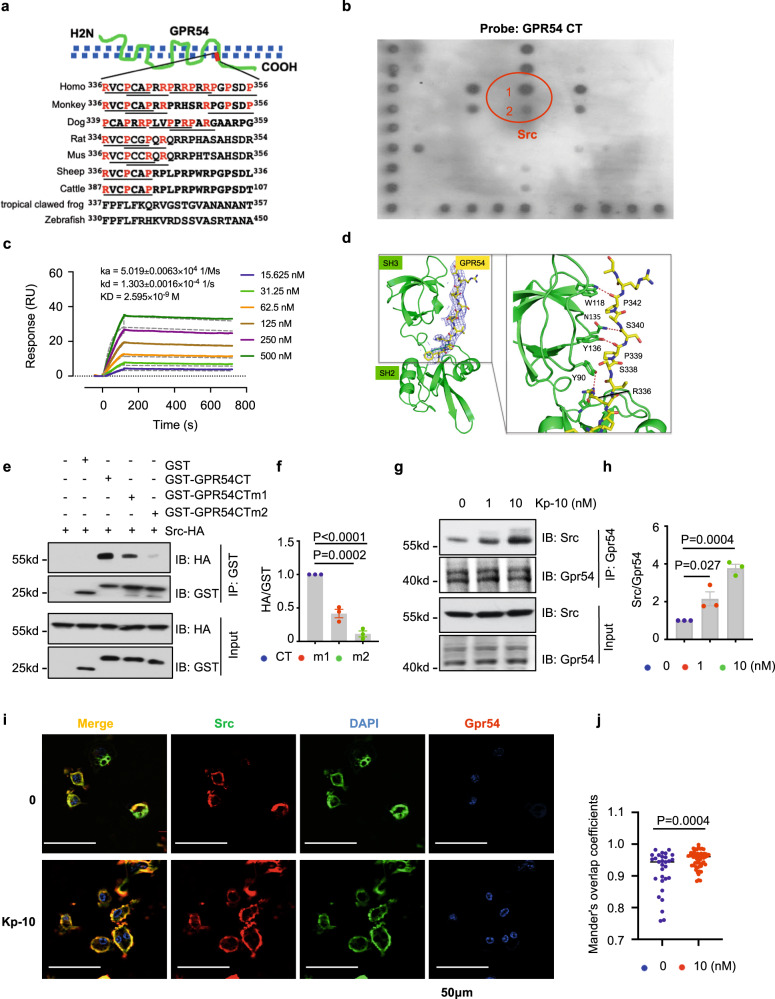
GPR54 recruited Src via the PR motif in GPR54 C-Terminus. **a** Sequence analysis of the PR motif in multiple species of GPR54 CT. **b** SH3 domain protein-binding array using the TranSignal™ SH3 Domain Array kit. The principal Src binding region is circled in red evident (1 Src; 2 the dilution of Src in half). **c** SPR binding-affinity measurement of Src and GPR54 CT. Src and GPR54 CT binding affinity is 2.6 nM (kinetic analysis, RUmax = 36.31, Chi = 1.08). **d** Crystallization structure analysis of the SH2-SH3 domain of Src and the human GPR54 ^336–356^ piptide including PR motif. GPR54 CT bound with the SH3 domain of Src mainly via the key residues including R336, P339 and P342. **e**–**h** IB analysis of total samples and GST pull-downs using GST proteins including GST, GST-GPR54 CT, GST-GPR54 CT m1 (R336A&P339A), GST-GPR54 CT m2 (delta 339–344) incubated separately with WCL of 293 T cells transfected with HA-Src (**e**) and quantification of protein levels (**f**), IB analysis of WCL and anti-GPR54 IP assays derived from RAW264.7 cells induced by Kp-10 for 20 min (**g**) and quantification of protein levels (**h**). **i**, **j** IF staining was carried out using pre-osteoclasts differentiated from BMMs with M-CSF (10 ng/ml) and RANKL (50 ng/ml) stimulation for 2 days, then treated with 10 nM Kp-10 for 20 min. Representative images of endogenous Gpr54 and Src were shown by total internal reflection fluorescence (TIRF) microscopy (**i**), and the Mander’s overlap coefficient (MOC) as a measure of colocalization in cells from multiple images taken randomly (**j**). Scale bar, 50 μm. Data represent means ± SEM. *P* values were determined by one-way ANOVA analysis (**f**, **h**) or two-tailed Student’s *t*-test (**j**). Representative results were obtained from at least three independent experiments. Source data are provided as a Source Data file.

By comparing multiple homologs of GPR54, it was observed that the SH3 binding motif was ubiquitous in the C-terminus (CT) of GPR54 in terrestrial vertebrates, and human GPR54 contains the most redundant SH3-binding sequence including two class I RxxPxxP motifs (GPR54^336–342^ and GPR54^350–356^) and two class II PxxPxR motifs (GPR54^339–344^ and GPR54^345–350^) (Fig. 2a). We screened SH3 domains to identify SH3 domain containing proteins that potentially bind to GPR54. C terminus of GPR54 (GPR54 CT) interacted with the SH3 domain of Src most obviously by SH3 domain proteins microarray assay (Fig. 2b). Of note, GPR54 CT dose-dependently bound with Src by surface plasmon resonance (SPR) analysis, and the binding affinity was calculated to be approximately 2.6 nM (Fig. 2c). We were not able to co-crystallize the SH3 domain or SH3-SH2 domains of Src with the whole CT of GPR54 or its PR motif, but a chimera construct embedding the PR motif (333–356) to the C-terminus of SH3-SH2 domains yielded crystals that diffracted to 3.54 Å. The structure reveals interactions formed between the SH3 domain and the N-terminal half of the PR motif, e.g., there are key hydrophilic interactions between GPR54 residues R336, P339, and P342, and SH3 residues Y190, Y136, and W118, respectively (PBD ID: 7YQE, Fig. 2d). Consistent with this structure, SPR showed that Src bound with the peptide of GPR54^336–356^ with binding affinity of 4.24 μM (Supplementary Fig. 3a), the binding was only slightly reduced for the N-terminal half of the PR motif (GPR54^333–344^, 28.27 μM, Supplementary Fig. 3b). Consistently, by pull-down assay, the R336A&P339A mutant of human GPR54 CT, which removed the N-terminal key proline and arginine, dramatically dampened the interaction of Src with GPR54 CT, and the △339–344 (deletion sign: △) mutant of GPR54 CT completely abolished the binding of Src with GPR54 CT (Fig. 2e, f).

Consistently, the interaction of Gpr54 with Src was enhanced after treatment of RAW264.7 cells with Kp-10 (Fig. 2g, h) and also in 293 T cells transfected with HA-Src and GPR54-Flag constructs (Supplementary Fig. 3c, d). In addition, immunofluorescence (IF) staining assay by total internal reflection fluorescence (TIRF) microscopy demonstrated that Src co-localized on the membrane with Gpr54 especially after Kp-10 stimulation in pre-osteoclasts (Fig. 2i, j), IgG, Src and Gpr54 only are showing as control (Supplementary Fig. 3e). These findings coherently showed that GPR54 bound with Src via PR motif after Kp-10 treatment.

### The phosphatase DUSP18 was recruited by GPR54 upon activation by the ligand Kp-10 via the PR motif in GPR54 C-Terminus

**Fig. 3:**
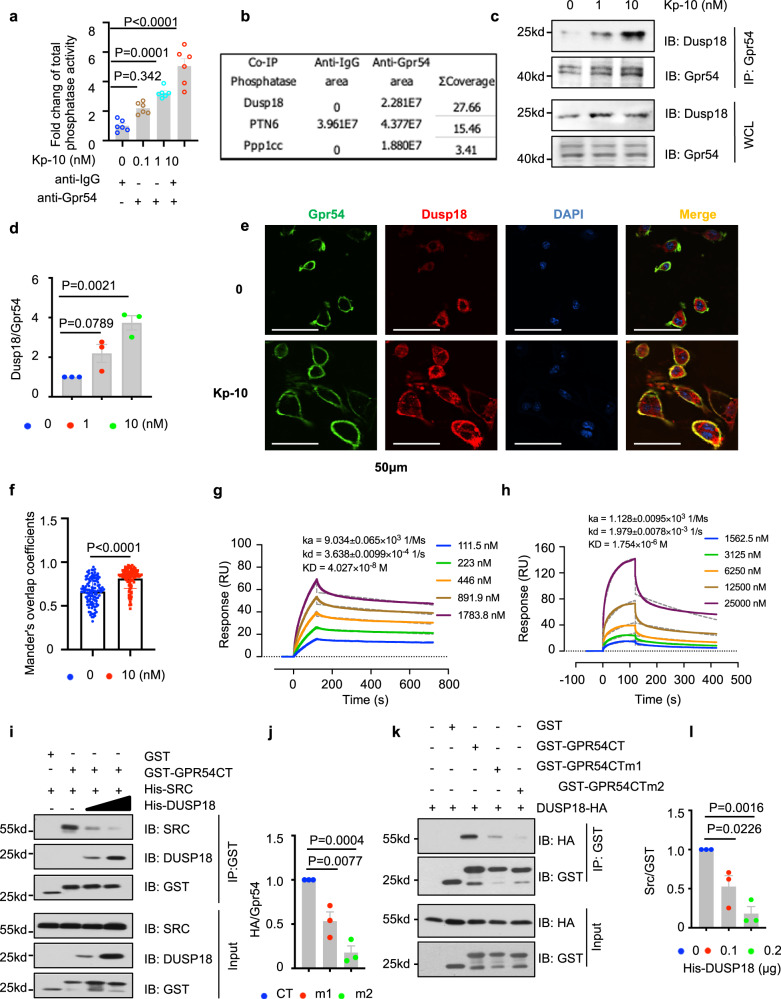
DUSP18 phosphatase was recruited by GPR54 after Kp-10 stimulation via the PR motif. **a**–**d** Anti-Gpr54 IP was performed on WCL derived from RAW264.7 cells treated with indicated dose of Kp-10 for 20 min. Beads were analyzed by phosphatase activity assay (**a**), mass spectrometry assay (**b**), IB analysis (**c**) and quantification of protein levels (**d**). **e**, **f** Protein co-localization was analyzed by IF staining in primary pre-osteoclast with 10 nM Kp-10 treatment for 20 min. Representative images of endogenous Gpr54 and Dusp18 were shown by TIRF microscopy (**e**), and MOC was used to quantify the degree of colocalization in cells from multiple images taken randomly (**f**). Scale bar, 50 µm. **g**, **h** binding affinity was measured by SPR binding analysis. The binding affinity of DUSP18 and human GPR54 CT was measured at 40 nM, RUmax = 68.22, Chi = 1.01 (**g**), DUSP18 and mouse Gpr54 ^336–342^ was measured at 1.8 µM, RUmax = 154.6, Chi = 3.42 (**h**). **i**–**l** IB and quantification of protein levels analysis of total samples and GST pull-downs using GST proteins including GST, GST-GPR54 CT, GST-GPR54 CT m1 (R336A&P339A), GST-GPR54 CT m2 (delta 339–344) incubated with WCL of 293 T cells transfected with DUSP18-HA (**i**) and quantification of protein levels (**j**), Total samples and GST pull-downs using His-SRC protein (0 or 0.1 μg) purified from Sf9 cells, His-DUSP18 (0, 0.1 or 0.2 μg), and GST proteins purified from *E. coli* (**k**) and quantification of protein levels (**l**). Data represent means ± SEM. *P* values were determined by one-way ANOVA analysis (**a**, **d**, **j**, **l**) or two-tailed Student’s *t*-test (**f**). Representative results were obtained from at least three independent experiments. Source data are provided as a Source Data file.

Our results above showed that Src phosphorylation was inhibited by Kp-10 /Gpr54. Next, we sought to determine whether one or more phosphatases co-immunoprecipitated with Gpr54 upon Kp-10 stimulation. Activities of total phosphatases co-immunoprecipitated with Gpr54 were enhanced by Kp-10 stimulation (Fig. 3a). In contrast to the phosphatases co-immunoprecipitated with GPR54 antibody, three increasing phosphatases including DUSP18, PTPN6 and PPP1CA were identified after Kp-10 stimulation by mass spectrometry analysis (Data are available via ProteomeXchange with identifier PXD038433) (Fig. 3b). Furthermore, Co-IP analysis of GPR54 CT with DUSP18, PTPN6 and PPP1CA were performed and the results showed that DUSP18 seemed to be a crucial tyrosin phosphatase which had the strongest interaction with GPR54 (Supplementary Fig. 3f, g). Notably, Western blotting showed that Dusp18, Kiss1 and Gpr54 were up-regulated during osteoclasts formation by Western blotting (Supplementary Fig. 4a, b). These results were confirmed by immunohistochemistry (IHC) assay in ovariectomy (ovx) -induced postmenopausal osteoporosis bone tissues comparing with that of sham-operated mice, and in giant cell tumor of bone (GCTB) characterized histologically by the presence of osteoclast-like giant cells (Supplementary Fig. 4c). Furthermore, to verify human anti-GPR54 antibody and anti-KiSS1 antibody in mouse, IF staining in Gpr54^+/+^ and Gpr54^−/−^ osteoclasts (Supplementary Fig. 5a) and Kiss1^+/+^ and Kiss1^−/−^ osteoclasts (Supplementary Fig. 5c), IHC assay in the femurs of Gpr54^+/+^ and Gpr54^−/−^ mice (Supplementary Fig. 5b) and Kiss1^+/+^ and Kiss1^−/−^ mice (Supplementary Fig. 5d), and immunoblots assay in Kiss1^+/+^ and Kiss1^−/−^ BMMs (Supplementary Fig. 5e) were performed.

Furthermore, Co-IP analysis also validated that Kp-10 dose-dependently induced the interaction of Dusp18 with Gpr54 in RAW264.7 cells (Fig. 3c, d) and in 293 T cells transfected with DUSP18-HA and GPR54-Flag (Supplementary Fig. 6a, b). IF staining assay by TIRF microscopy showed that Dusp18 colocalized with Gpr54 in the plasma membrane especially after treatment of Kp-10 in pre-osteoclasts (Fig. 3e, f).

By SPR analysis, we observed that human GPR54 CT dose-dependently bound with DUSP18 with a binding affinity of 40 nM (Fig. 3g). Similarly, we then used synthesized mouse Gpr54 peptides to test the binding with Dusp18. The peptide of Gpr54^336–342^ which corresponds to the RxxPxxR motif bound to Dusp18 with a binding affinitiy of 1.8 μM (Fig. 3h), whereas the right-shifted peptide of Gpr54^339–344^ which corresponds to the PxxRxR motif generated an unfitted SPR curve when titrate with Dusp18 (Supplementary Fig. 6c). Reversely, a human GPR54 CT double point mutation R336A&P339A which damaged the R^336^xxPxxP^342^ motif and the P^339^xxPxR^344^ motif dampened its interaction with DUSP18, and a truncation (△339–344) which destroyed the two SH3 binding motifs in the left hand also abolished the binding with DUSP18 (Fig. 3i, j). Furthermore, GST pull-downs using GST proteins including GST, GST-GPR54 CT, GST-GPR54 CT (R350&P353) incubated separately with WCL of 293 T cells transfected with HA-Src or HA-DUSP18 were performed that GST-GPR54 CT (R350&P353) showed no loss of binding (Supplementary Fig. 6d, e), which also significantly strengthen the arguments for selectivity for the first SH3 binding motif. Therefore, GPR54 interacted with DUSP18 also via the left-hand side of the PR motif in GPR54 CT. Of note, by competitive pull-down assay, we found that DUSP18 competed with Src for binding to GPR54 CT (Fig. 3k, l). Hence, these results coherently supported that the GPR54 CT recruited both DUSP18 and Src via the PR motifs upon Kp-10 stimulation.

### Src was dephosphorylated via DUSP18 when GPR54 was activated by its ligand Kp-10

**Fig. 4:**
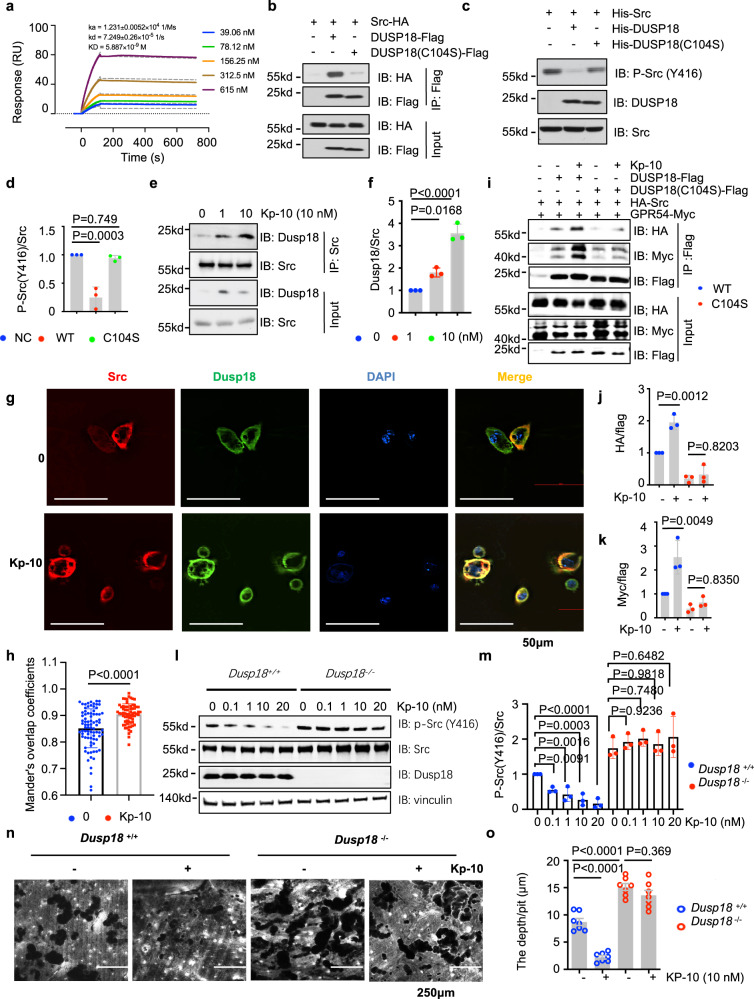
Src was dephosphorylated via DUSP18 following GPR54 activation by Kp-10. **a** The binding affinity of DUSP18 and Src was measured at 5.9 nM (RUmax = 130.6, Chi = 3.42) by SPR binding analysis. **b**–**f** IB and quantification of protein co-IP levels. Anti-Flag IP derived from 293 T cells transfected with HA-Src and either DUSP18-Flag or DUSP18 (C104S)-Flag constructs with or without Kp-10 treatment for 20 min were lysed and subjected to anti-FLAG IP (**b**). Phosphatase reaction products using His-DUSP18 and His-DUSP18 (C104S) proteins purified from *E. coli* and SRC proteins purified from Sf9 insect cells (**c**) and quantification of protein levels (**d**). Anti-Src IP derived from RAW264.7 cells treated with or without Kp-10 treatment for 20 min were lysed and subjected to anti-Src IP (**e**) and quantification of protein levels (**f**). **g**, **h** Src and Dusp18 co-localization was analyzed by IF staining in primary pre-osteoclasts treated with 10 nM Kp-10 treatment for 20 min. Representative images of endogenous Dusp18 and Src were shown by TIRF microscopy (**g**), and MOC was used to quantify the degree of colocalization in cells from multiple images taken randomly (**h**). Scale bar, 50 µm. **i**–**m** IB and quantification of protein levels analysis. Anti-Flag IP derived from 293 T cells transfected with HA-Src, GPR54-myc, and either DUSP18-Flag or DUSP18 (C104S)-Flag constructs after Kp-10 treatment for 20 min (**i**) and quantification of protein levels (**j**, **k**). WCL derived from WT and *Dusp18*^−/−^ BMMs treated with or without Kp-10 for 20 min (**l**) and quantification of protein levels (**m**). **n**, **o** WT and *Dusp18*^−/−^ BMMs were cultured in vitro on dentin slices, and after incubation with M-CSF (10 ng/ml) and RANKL (50 ng/ml) for 5–7 days, the pits formed by osteoclast absorption activity were scanned by confocal microscopy (XY and z sections). Scale bars, 125 µm (**n**), and pits depth were quantified by confocal microscopy (**o**). Data represent means ± SEM. *P* values were determined by one-way ANOVA analysis (**d**, **f**, **j**, **k**, **m**, **o**) or by two-tailed Student’s *t*-test (**h**). Representative results were obtained from at least three independent experiments. Source data are provided as a Source Data file.

By SPR analysis, we found that DUSP18 can also interacted with Src with a binding affinity of 5.9 nM (Fig. 4a). Interestingly, in contrast to WT-DUSP18, a mutation in the catalytic center of DUSP18 (C104S) eliminated the interaction with Src by Co-IP analysis (Fig. 4b, and Supplementary Fig. 6f). Purified WT-DUSP18 potently reduced the phosphorylation of Src (Y416) purified from Sf9 insect cells in vitro, but the enzymatically dead DUSP18 (C104S) mutant had no activity (Fig. 4c, d). The interaction of Dusp18 with Src was enhanced after Kp-10 stimulation in RAW264.7 cells by Co-IP analysis (Fig. 4e, f). Consistent with this data, IF staining showed that Dusp18 colocalized with Src in the plasma membrane especially after treatment with Kp-10 in pre-osteoclasts (Fig. 4g, h).

Notably, the complex formation of the heterotrimer including DUSP18, Src, and GPR54, was enhanced after Kp-10 stimulation in 293 T cells transfected with HA-Src, GPR54-Myc, and DUSP18-Flag (WT), but not when the catalytic center of DUSP18 was killed by introducing DUSP18 (C104S) mutation (Fig. 4i–k). In keeping with this finding, Kp-10 blocked Src phosphorylation in WT pre-osteoclasts but failed to do so in *Dusp18*-depleted BMMs (Fig. 4l, m). Therefore, bone resorption was suppressed in *Dusp18* WT osteoclasts but not in *Dusp18*^−/−^ osteoclasts as measured by the pit formation assay (Fig. 4n) and quantitation of the pit depth (Fig. 4o). Furthermore, bone resorption enhanced by *Dusp18* deletion was rescued upon ectopic expression of Dusp18 or knockdown of Src (Supplementary Fig. 6g, h). Phosphorylation of Src was enhanced by WB599 in WT BMMs but not in Dusp18^−/−^ BMMs (Supplementary Fig. 6i, j). These findings supported the notion that Dusp18 was a phosphatase of Src in this experimental setting, and Src was dephosphorylated via Dusp18 when Gpr54 was activated by Kp-10. Therefore, Kp-10/Gpr54 signaling suppresses osteoclast formation and bone resorption at least partially through Src dephosphorylation by its phosphatase Dusp18.

### Kp-10/Gpr54 upregulated the expression of Dusp18 and recruited both active Src and its phosphatase Dusp18

**Fig. 5:**
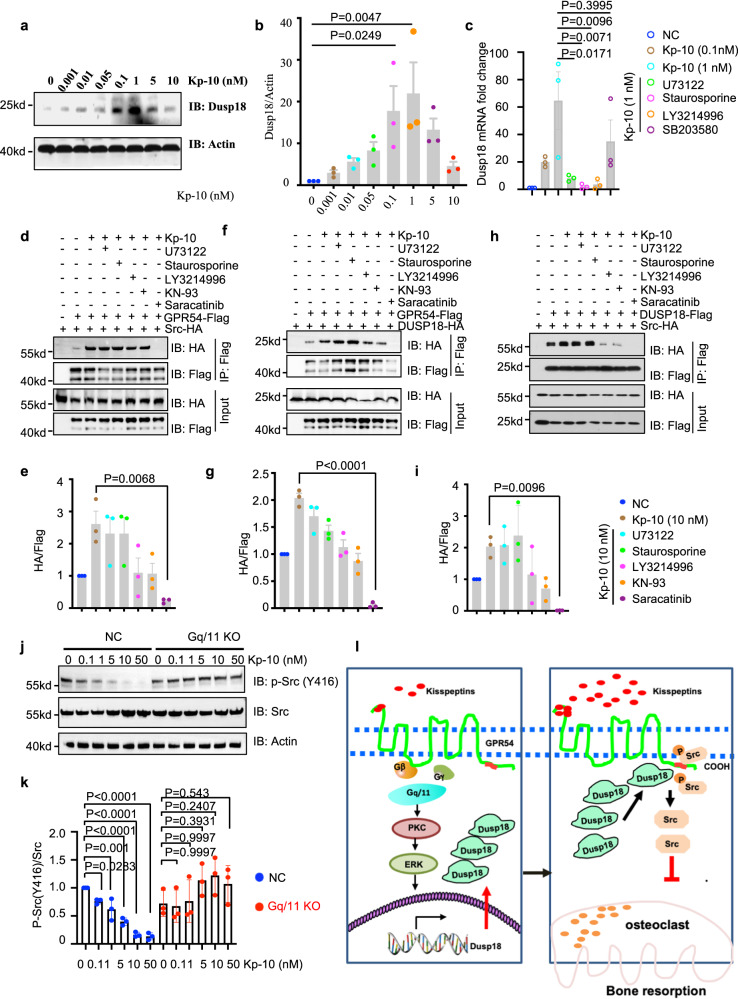
Kp-10/GPR54 upregulated the expression of Dusp18 and recruited both active Src and its phosphatase Dusp18. **a**, **b** IB analysis of lysates from RAW264.7 with indicated dose of Kp-10 treatment for 1 h (**a**) and quantification results (**b**). **c** Quantitative PCR of *Dusp18* mRNA in RAW264.7 cells treated with inhibitors of PLC (U73122, 10 µM), PKC (Staurosporine, 0.25 µM), ERK (LY3214996, 20 µM), and P38 (SB203580, 10 µM) for 1 h, and then incubated with indicated dose of Kp-10 for 30 min. **d**–**i** Anti-Flag IP of lysates derived from 293 T cells transfected with indicated constructs and treated with PLC inhibitor (U73122, 10 µM), PKC inhibitor (Staurosporine, 0.25 µM), ERK inhibitor (LY3214996, 10 µM), Ca2+/CaMKII inhibitor (KN-93, 10 µM), and Src inhibitor (Saracatinib, 10 µM) for 1 h and then stimulated with 10 nM Kp-10 for 20 min. Anti-Flag IP of lysates derived from 293 T cells transfected with GPR54-flag and Src-HA constructs (**d**) and quantification results (**e**). Anti-Flag IP of lysates derived from 293 T cells transfected with GPR54-flag and DUSP18-HA constructs (**f**) and quantification results (**g**). Anti-Flag IP of lysates derived from 293 T cells transfected with Src-HA, and DUSP18-flag constructs (**h**) and quantification results (**i**). **j**, **k** IB analysis of lysates derived from WT BMMs and Gq/11 KO BMMs with or without Kp-10 treatment for 1 h (**j**) and quantification of results (**k**). **l** Working model of Kp-10 /Gpr54 mediated Src dephosphorylation. Low dose of Kp-10 (0.1, 1 nM) induced the expression of Dusp18 obviously but not by 10 nM Kp-10, which is dependent on Gq/11 signaling. However, both active Src and DUSP18 were recruited by GPR54 through the PR motif in GPR54 CT along with the increase of Kp-10 dose (1, 10 nM). Therefore, phosphorylation of Src was dose dependently suppressed when GPR54 was activated by Kp-10. Data represent means ± SEM. *P* values were determined by one-way ANOVA analysis (**b**, **c**, **e**, **g**, **i**, **k**). Representative results were obtained from at least three independent experiments. Source data are provided as a Source Data file.

Interestingly, Kp-10 (0.1, 1 nM) obviously upregulated the expression of Dusp18 as revealed by WB analysis (Fig. 5a, b) in RAW264.7 cells. Notably, the expression of Dusp18 was inhibited by the inhibitor of Gq/11 downstream signals including PKC (Staurosporine) and ERK (LY3214996) but not by the inhibitor of PLC (U73122), or P38 (SB203580) by qPCR assay (Fig. 5c). Additionally, the protein level of Dusp18 cannot be obviously up-regulated by Kp-10 when ERK1/2 was knocked down by siRNA in RAW264.7 cells (Supplementary Fig. 7a, b). These results supported the notion that Kp-10/Gpr54 upregulates the expression of Dusp18 at least partially through the Gq/11-PKC-ERK signaling pathway.

The interaction of GPR54 with Src (Fig. 5d, e), GPR54 with DUSP18 (Fig. 5f, g), and Src with DUSP18 (Fig. 5h, i) in 293 T cells transfected with indicated constructs and induced by Kp-10 were all obvioulsly dampened by Src kinase inhibitor (Saracatinib), but not by the inhibitors of Gq/11 downstream signals including PLC inhibitor (U73122), PKC inhibitor (Staurosporine), ERK inhibitor (LY3214996), and Ca2+/ CaMKII inhibitor (KN-93). Furthermore, the binding of GPR54 with DUSP18 promoted by Kp-10 was blocked upon Src siRNA knockdown in 293 T cells transfected with DUSP18-HA and GPR54-Flag (Supplementary Fig. 8a, b). However, the interaction of GPR54 with DUSP18 was still induced by Kp-10 when ERK1/2 was depleted via siRNA knockdown (Supplementary Fig. 8c, d). In summary, active Src and its phosphatase Dusp18 were recruited by Gpr54 upon Kp-10 stimulation.

Upon ligand activation, GPCRs trigger pathways via both G proteins and β-arrestins^30^. Therefore, we explored whether Src dephosphorylation mediated by GPR54 was dependent on Gq/11, β-arrestin-1/2 or not. IB analysis showed that Kp-10 inhibited the phosphorylation of Src in WT BMMs, but failed to have a significant effect in Gq/11 KO BMMs (Fig. 5j, k). Futhermore, the phosphorylation of Src was still suppressed (Supplementary Fig. 9a, b), and the interaction of Gpr54 with Src was also enhanced (Supplementary Fig. 9c, d) after Kp-10 stimulation in *Arrb1* and *Arrb2* double-knockout MEFs. Moreover, Kp-10 still blocked osteoclast formation in *Arrb1*^*−/−*^ BMMs (Supplementary Fig. 9e, f) and in *Arrb2*^*−/−*^ BMMs (Supplementary Fig. 9g, h). Taken together, our findings demonstrated that the expression of Dusp18 was obviously upregulated by 0.1, 1 nM Kp-10 but not by 10 nM Kp-10. Furthermore, both active Src and Dusp18 were significantly recruited and the phosphorylation of Src was blocked along with the increase of Kp-10 dose, which is dependent on Gq/11 signaling but not relies on β-arrestin-1/2 (Fig. 5l).

### Mice deficient in *Kiss1*, *Gpr54*, or *Dusp18* exhibited bone loss and osteoclast hyperactivation

**Fig. 6:**
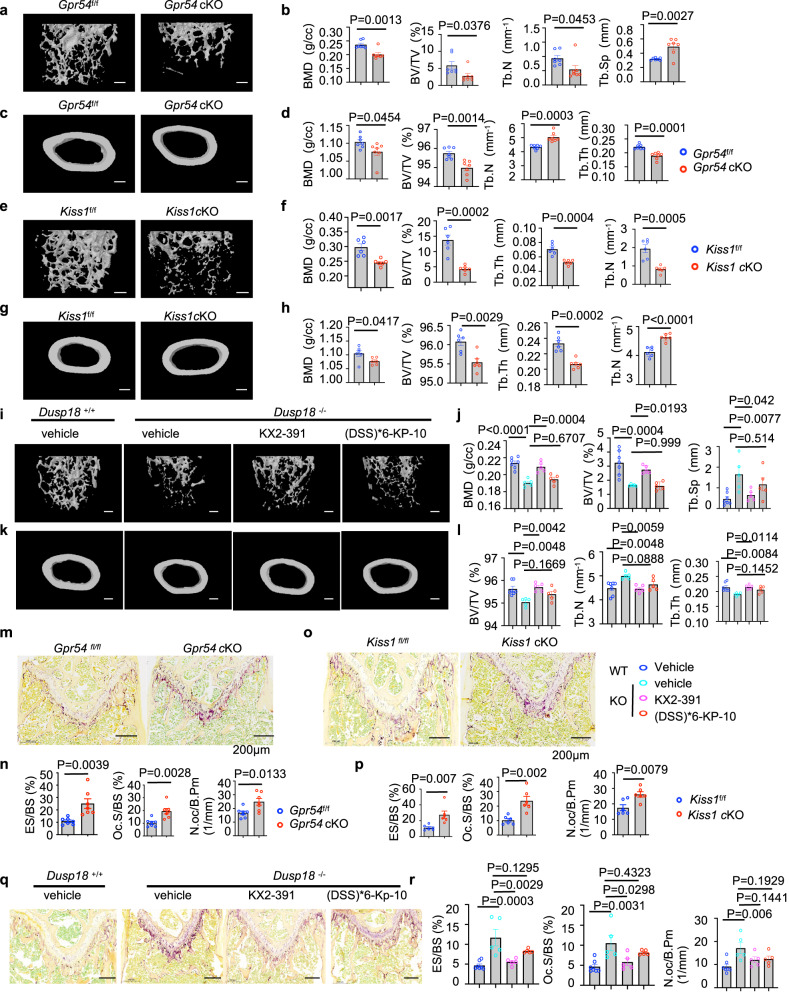
Osteoclast conditional knockout *Gpr54* mice (Gpr54 cKO) and *Kiss1* mice (*Kiss1* cKO), whole-body knockout mice of Dusp18 (*Dusp18*^−/−^) mice exhibited bone loss and osteoclast hyperactivation. **a**, **c**, **e**, **g**, **i**, **k** Representative micro-CT images of femoral trabecular bone and cortical bone. 4-month-old WT and *Gpr54* cKO mice (*n* = 7 per group including 4 female and 3 male mice) (**a**, **c**), 4-month-old WT and *Kiss1* cKO mice (*n* = 6 per group including 3 female and 3 male mice) (**e**, **g**) and 8-week-old WT and Dusp18^−/−^ mice with or without treatment of KX2-391 or (DSS)*6-KP-10 (*n* = 7 for WT mice including 3 female and 4 male mice, *n* = 5 for each other group including 2 female and 3 male Dusp18^−/−^ mice) (**i**, **k**). Scale bar, 500 µm. **b**, **d**, **f**, **h**, **j**, **l** Bone parameters analysis of femurs from the mice above. 2-month-old WT and *Gpr54* cKO mice (**b**, **d**), 4-month-old WT and *Kiss1* cKO mice (**f**, **h**), 4-month-old and Dusp18^−/−^ mice (**j**, **l**). BMD bone mineral density, BV/TV bone volume as a fraction of total bone volume, Tb.Th trabecular thickness, Tb.N trabecular number, Tb.Sp trabecular separation. **m**, **o**, **q** Representative TRAP staining images of femurs from the mice above. 4-month-old and *Gpr54* cKO mice (**m**), 4-month-old and *Kiss1* cKO mice (**o**) and 4-month-old and Dusp18^−/−^ mice (**q**). Scale bar, 200 µm. **n**, **p**, **r** Osteoclast parameters analysis of femurs from the mice above. 4-month-old WT and *Gpr54* cKO mice (**n**), 4-month-old and *Kiss1* cKO mice (**p**), 4-month-old and Dusp18^−/−^ mice (**r**). Data represent means ± SEM. *P* values were determined by two-tailed Student’s *t*-test (**b**, **d**, **f**, **h**, **n**, **p**) or one-way ANOVA analysis (**j**, **l**, **r**). Representative results were obtained from at least three independent experiments. Source data are provided as a Source Data file.

Osteoclast specific condition knockout of *Gpr54 (Gpr54* cKO) mice, or *Kiss1 (Kiss1* cKO) mice were obtained by crossing *Gpr54* ^f/f^ or *Kiss1*^f/f^ mice with LysCre mice. The targeting strategies of *Gpr54* ^f/f^ (Supplementary Fig. 10a) and *Kiss1*^f/f^ (Supplementary Fig. 10b) were shown. Comparing with the indicated wild-type mice, elisa assay showing the same level of testosterone, luteinizing hormone (LH) and follicle-stimulating hormone (FSH) in *Gpr54* cKO mice (Supplementary Fig. 10c, d), and in *Kiss1* cKO mice (Supplementary Fig. 10e, f). However, testosterone, LH and FSH obviously declined in whole -body Gpr54 knockout mice (Gpr54^−/−^, Supplementary Fig. 11a, b) and in whole -body Kiss1 knockout mice (Kiss1^−/−^, Supplementary Fig. 11c, d). Bone loss was observed in *Gpr54* cKO mice (Fig. 6a–d), *Kiss1* cKO mice (Fig. 6e–h) and the whole body knockout of *Gpr54 mice* (Supplementary Fig. 11e–h), *Kiss1* mice (Supplementary Fig. 11i–l), and *Dusp18* mice (Fig. 6i–l) by micro-CT and bone parameters analysis. Consistently, *Dusp18* deletion induced bone mass was efficiently rescued by treatment of Src inhibitor KX2-391 but not by (DSS)*6-KP-10 (Fig. 6i–l).

Osteoclasts were hyperactivated in the femurs of *Gpr54* cKO mice (Fig. 6m, n), *Kiss1* cKO mice (Fig. 6o, p), the calvaria of *Kiss1*^−/−^ mice and *Gpr54*^−/−^*mice* (Supplementary Fig. 12a, b), and the femurs of *Dusp18*^−/−^ *mice* (Fig. 6q, r) by TRAP staining and osteoclast parameters analysis. Furthermore, *Dusp18* deletion induced osteoclast activity was efficiently rescued by the Src inhibitor KX2-391 but not by (DSS)*6-KP-10 (Fig. 6q, r). Taken together, our data provide strong evidence to support a bone-protective role of Kp-10/Gpr54 by negatively modulating osteoclast activities in vivo.

Physiologically, a stable bone mass was maintained by the balance between bone resorption by osteoclasts and bone formation by osteoblasts, and osteoclastogenesis were supported by osteoblast-derived RANKL and M-CSF^4^. Bone formation by osteoblast was also enhanced in *Gpr54*^−/−^ *mice* (Supplementary Fig. 13a, b), *Kiss1*^−/−^ mice (Supplementary Fig. 13c, d) but not in *Gpr54* cKO (Supplementary Fig. 13e, f) and *Kiss1* cKO mice (Supplementary Fig. 13g, h) by double calcian labeling assay. Goldner’s Masson trichrome staining showed more osteoid/hypomineralized areas (stained red) in *Gpr54*^−/−^ *mice* (Supplementary Fig. 13i, j), in *Kiss1*^−/−^ mice (Supplementary Fig. 13k, l) and in *Dusp18*^−/−^ mice (Supplementary Fig. 14a, d). Consistently, osteoblasts differentiation were promoted when *Gpr54* or *Kiss1* was deleted as determined by ALP staining (Supplementary Fig. 15a) and von Kossa staining (Supplementary Fig. 15b). Furthermore, osteoblast differentiation was dose-dependently suppressed upon Kp-10 stimulation (Supplementary Fig. 15c, d).

### Kp-10 ameliorated OVX-induced bone loss

**Fig. 7:**
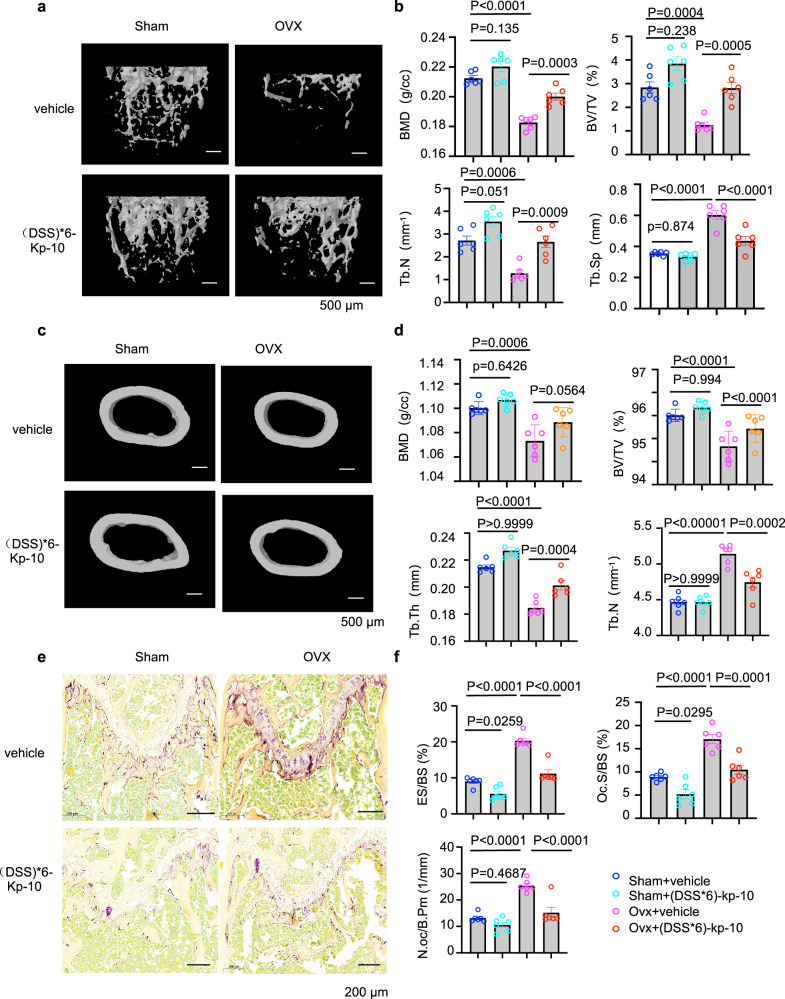
Kp-10 ameliorated OVX-induced bone loss. **a**, **c** Representative micro-CT images of femoral trabecular bone (**a**) and cortical bone (**c**). Female C57 mice treated with vehicle (Sham) or 50 nmol/kg (DSS)*6-Kp-10, ovariectomized mice (OVX) treated with vehicle or 50nmol/kg (DSS)*6-Kp-10 twice one week by intraperitoneal injection (*n* = 6 per group). Scale bar, 500 µm. **b**, **d** Parameter analysis of femurs from the mice above. BMD bone mineral density, BV/TV bone volume as a fraction of total bone volume, Tb.Th trabecular thickness, Tb.N trabecular number, Tb.Sp trabecular separation. **e** Representative TRAP staining images of femurs from Fig. 7a. **f** Histomorphometry analysis of the femurs from Fig. 7a. Oc.S/BS osteoclast surface per bone surface, N.Oc/B.Pm osteoclast bone surface density, ES/BS eroded surface per bone surface. Data represent means ± SEM. *P* values were determined by one-way ANOVA analysis (**b**, **d**, **f**). Representative results were obtained from at least three independent experiments. Source data are provided as a Source Data file.

Our data above showed that Kp-10/Gpr54 negatively regulated osteoclast activity via Src dephosphorylation by Dusp18. Therefore, we explored whether Kp-10 could ameliorate bone loss in vivo. In ovariectomized mice, after intravenous injection of Kp-10 (1, 10 nmol/kg) and bone targeting Kp-10 ((DSS)*6-Kp-10) (1, 10 nmol/kg) twice per week for two months, we observed that mice with 1 or 10 nmol/kg (DSS)*6-Kp-10 showed better bone protective effect than 1 or 10 nmol/kg Kp-10 correspondingly as determined by Von Kossa staining (Supplementary Fig. 16a) and parameters of trabecular bone analysis (Supplementary Fig. 16b). Elisa assay showing the same level of FSH and LH after treatment with bone targeting Kp-10 ((DSS)*6-Kp-10) (10 nmol/kg) twice per week for two months in Sham mice and in OVX mice comparing with the indicated mice treated with vechile respectively (Supplementary Fig. 16c). Furthermore, bone mass increased in sham-operated mice as well as in OVX mice after treatment with 50 nmol/kg (DSS)*6-Kp-10 by micro-CT analysis (Fig. 7a–d). Consistent with these results, 50 nmol/kg (DSS)*6-Kp-10 treatment suppressed osteoclast activation both in sham-operated and OVX mice by TRAP staining (Fig. 7e) and the measurement of osteoclast parameters (Fig. 7f). Thus, these data coherently suggested that the Kp-10/Gpr54 signaling axis played a bone protective role both in vitro and in vivo via blocking osteoclastic bone resorption.

## Discussion

Targeted disruption of the *Src* gene in mice leads to a predominant phenotype of osteopetrosis due to a defect in osteoclast function, which revealed that Src is essential for bone resorption by osteoclasts^5,14^. Therefore, Src has been considered as a candidate drug target for anti-osteoporosis. Understanding the molecular mechanisms of Src is essential for a complete molecular-level understanding of bone resorption and for designing novel therapeutic approaches for treating bone disease. In this study, we identified Dusp18 as a phosphatase of Src in osteoclasts. Both active Src and Dusp18 were recruited by Gpr54 via the PR motif in the C terminus of Gpr54 upon activation by its ligand Kp-10. Therefore, we revealed a new mechanism of bone resorption: Kp-10/Gpr54 negatively regulated bone resorption mainly via Src dephosphorylation by Dusp18.

Osteoclasts emerged during the evolution from aquatic to terrestrial life. In adaptation to terrestrial life, the solid bones of aquatic vertebrates were changed by osteoclasts to a hollow architecture that has a superior strength-to-weight ratio^40,41^. In the C terminus of GPR54, we found a conserved PR motif that emerged in terrestrial animals (Fig. 2a). Notably, aquatic vertebrates like zebrafish or tropical clawed frogs contain no PR motif in the corresponding position of each sequence, suggesting that the PR motif may have been acquired during the evolution from aquatic creatures to land animals and may be involved in some functions unique to terrestrial vertebrates. Our results showed that Kp-10/Gpr54 suppressed Src phosphorylation by recruitment of both active Src and Dusp18 via the PR motif in the GPR54 C terminus. Hence, the emergence of the PR motif in the GPR54 C terminus in terrestrial animals may have been an important adaptation for life on land by modulating Src activity in osteoclasts.

It is estimated that 30 to 50% of marketed drugs act directly on G protein-coupled receptors (GPCRs)^23,24^, making GPCRs the most important class of drug targets. A typical example is the calcitonin receptor (CTR), which negatively regulates osteoclast resorption. Calcitonin, the ligand of CTR, has been in clinical use for conditions involving accelerated bone turnover, including Paget’s disease and osteoporosis^42,43^. Our results coherently suggested that treatment with a bone targeting Kp-10, (DSS)*6-Kp-10, showed bone protection in the OVX-induced bone loss model by inhibiting osteoclast activity (Fig. 7). This suggests a potential value for targeting GPR54 in diseases characterized by bone loss.

GPCR signals are transduced through two different mechanisms with agonist dosage acting as the switch. For example, β2-adrenergic receptors (β2-ARs) activate the mitogen-activated protein kinase through Gαs at low concentrations of an agonist, whereas at higher concentrations Src was activated directly by β2-ARs independent of Gαs and β-arrestins. This dosage-dependent switch of GPCRs signaling has significant implications for GPCR intrinsic properties and desensitization^28^.Src is activated by GPCRs through different ways including direct interaction via its SH3 binding motif in the intracellular domain^25–28^, or indirect activation of Src via arrestins^30^ or Gα_s/i_^29^. Gαs and Gαi did stimulate autophosphorylation of Src (Tyr416) directly, but Gα_q_, Gα_12_, and Gβγ cannot. In this study, phosphatase Dusp18 was upregulated at low concentration of Kp-10. Both active Src and phosphatase Dusp18 were recruited by the PR motif in the Gpr54 C terminus after treatment with dosage of Kp-10. Kp-10/Gpr54 mediated suppression of Src phosphorylation is dependent on Gq/11 signaling but not relies on beta-arrestin (Fig. 5h, i). However, how Gq/11 takes part in the process is still unclear, which warrants future in-depth investigation.

Kisspeptins/Gpr54 signaling in hypothalamus neurons is the switch of puberty by governing hormone release via the hypothalamic-pituitary-gonadal axis. Gonadotropin-releasing hormone (GnRH) is stimulated by Kisspeptins/Gpr54 and then acts on the anterior pituitary triggering the release of LH, and FSH. In addition, FSH and LH prompt the ovaries to begin producing the hormone estrogen and work together to get the testes to begin producing testosterone^31,32^. Both estrogen and androgen play a role in bone protection^44–46^. Our results showed that bone loss happened in mice with the whole-body knockout of *Kiss1* or *Gpr54*, and both osteoblasts and osteoclasts were hyperactivated. This is reasonable because osteoclast activation needs to be supported by osteoblast-derived cytokines including M-CSF and RANKL. Consistent with our findings, Herber and Ingraham et al. found that ablating estrogen receptor alpha (ERα), thus removing the negative regulation of Kiss1 expression by ERα in the medial basal hypothalamus (*Esr1*^Nkx2-1Cre^), promoted bone mass, which revealed that Kiss1 also played a bone protective role as described in this study^44^. However, both osteoblasts and osteoclasts were activated in *Esr1*^Nkx2-1Cre^ mice, which was inconsistent with the phenotype of osteoblasts and osteoclasts in the whole-body knockout of *Kiss1* or *Gpr54* mice^44^. Bone loss induced by the whole-body knockout of *Kiss1* or *Gpr54*, and the *Esr1*^Nkx2-1Cre^ induced bone mass increase are supposed to be indirectly affected by hormones from the hypothalamic-pituitary-gonadal axis. Our results showed that *Kiss1* or *Gpr54* are highly expressed in osteoclasts (Supplementary Fig. 1c, Supplementary Fig. 4), osteoclast-specific knockout of *Kiss1* or *Gpr54* exhibited osteoclast-hyperactivation-mediated bone loss (Fig. 6a–h), and bone targeting Kp-10, (DSS)*6-Kp-10 showed better effects than Kp-10 on bone protection in osteoclast overactivated mice model (Supplementary Fig. 15a, b). This suggests osteoclastic Kp-10/Gpr54 played an important role in bone protection via suppression of osteoclast bone resorption.

Typically, GPCR activation with increasing of ligand concentration generates different signalings and phenotypes^45^. In the working model of Kp-10/Gpr54-mediated suppression of Src phosphorylation, the expression of Dusp18 was obviously upregulated by 0.1, 1 nM Kp-10 but not by 10 nM Kp-10 (Fig. 5a). Howevere, Src and Dusp18 were more significantly recruited by Gpr54 upon activation by 10 nM Kp-10 (Fig. 2i, j, Fig. 3c, d, Fig. 4e, f, Fig. 5d–i), and Src phosphorylation was more obviously blocked by 10 nM Kp-10 (Fig. 1c, d, Fig. 4l–m, Fig. 5j, k, Supplementary Fig. 1a, b). Furthermore, we found that *Kiss1* or *Gpr54* deletion enhanced osteoblast differentiation in vivo (Supplementary Fig. 12) and in vitro (Supplementary Fig. 13a, b). Consistent with this, Kp-10 effectively suppressed osteoblast differentiation at 0.01 nM and 0.1 nM (Supplementary Fig. 13c, d). However, Son et al. described that KP-10 stimulated osteoblast differentiation in vitro at 5 and 50 μM^46^, which was not consistent with the phenotype generated by *Kiss1* or *Gpr54* knockout.

## Methods

### Reagents and antibodies

The antibodies used in this study are listed in Supplementary Table 1. Recombinant mouse M-CSF protein (# 416-ML) and Recombinant mouse RANKL (462-TEC) were purchased from R&D systems. Peptides were from GL Biochem (Shanghai) LTD. Staurosporine (S1421), KN-93(S6787), U73122 (S8011), Saracatinib (S1006), LY3214996(S8534), SB203580 (S1076) were from Selleck.Puromycin (P8833), Pierce™ GST agarose (20211) and Pierce™ Protein A/G plus agarose (20423) were purchased from Thermo Fisher, USA. anti-HA-beads (IP0010) and Anti-FLAG-beads (F2426) were obtained from Sigma-Aldrich, USA.

### Cell culture

293 T, MEFs, BMMs and RAW264.7 cells were grown in Dulbecco’s Modified Eagle Medium (DMEM) supplemented with 10% fetal bovine serum, 100 units/ml penicillin, and 100 mg/ml streptomycin. Sf9 cells were grown in GIBCO® insect culture media supplemented with 10% fetal bovine serum (FBS), 100 units/ml penicillin, and 100 mg/ml streptomycin. Arrb1 and Arrb2 double knockout MEFs were a gift from Dr. Robert J. Lefkowitz (Duke University). All cell lines were tested to be negative for mycoplasma contamination.

### Transfections

Transfection of plasmid in 293 T cells was performed using PEI (Polysciences), siRNA was done in Raw264.7 cells and in 293 T cells with Lipofectamine™ LTX (Thermo Fisher) according to the manufacturer’s instructions.

### Lentivirus generation

To generate lentivirus, 293 T cells were plated in 10-cm dishes and transfected with lentiviral plasmids and the lentiviral packaging plasmids pMD2.G and psPAX2 at a ratio of 1:0.5:1 after transfection for 48–72 h, the supernatant was collected using filtered (0.22 μm filter, Millipore, USA).

### Kp-10 modification

Modified Kp-10 (D-Tyr–Asn-(D-Trp)-Asn-Ser-Phe-(azaGly)-Leu-Arg (Me)-Phe-NH2) was designed as previously described^47^, which showed not only high metabolic stability but also excellent GPR54 agonistic activity. Bone targeting Kp-10 (Asp-Ser-Ser-Asp-Ser-Ser-Asp-Ser-Ser-Asp-Ser-Ser-Asp-Ser-Ser-Asp-Ser-Ser- D-Tyr–Asn-(D-Trp)-Asn-Ser-Phe-(azaGly)-Leu-Arg (Me)-Phe-NH2) was designed using six repetitive sequences of aspartate, serine, and serine, which is a bone surface-targeting delivery system as previously described^38^.

### Site-directed mutagenesis

Site-directed mutagenesis to generate various DUSP18 and C-terminus of GPR54 mutants was performed using the QuikChange XL Site-Directed Mutagenesis Kit (Agilent) according to the manufacturer’s instructions.

### SH3 domain protein-binding array

SH3 domain protein-binding array was performed using TranSignal™ SH3 Domain Array kit (Cat. #MA3010). The kit was purchased from Panomics, and probed using GST-GPR54 CT. Purified different SH3 domain-containing proteins including the Src kinase family were spotted in duplicate on the membrane filter and tested for their binding to GST-GPR54 CT. Briefly, membrane filters were incubated with 30 mg/ml GST-GPR54 CT protein overnight at 4 °C. After washing the membrane, the GST antibody was incubated overnight at 4 °C. Protein binding was visualized by incubation with an HRP-conjugated antibody. Spots with stronger intensities indicated a higher binding affinity of GST-GPR54 CT with the SH3 domain-containing protein (s). SH3 domain proteins in the array were summarized in Supplementary Table 3.

### Surface plasmon resonance (SPR)

SPR was determined using a Biacore T200 instrument (GE). SRC or DUSP18 protein was immobilized on the sensor chip (CM5) using the amine-coupling method according to standard protocols. Immobilization was performed according to the manufacturer’s recommendations. SRC protein (64 ug/mL, pH5.0) or DUSP18 protein (25 ug/mL, pH7.4) was diluted in 10 mM acetate buffer or 10 mM PBS buffer. Sh2-sh3 (100 ug/mL, pH5.0) was diluted in 10 mM acetate buffer. Immobilization was performed according to the manufacturer’s recommendations. The kinetics and affinity assay were examined at 25 °C at a flow rate of 30 µl/minute using PBS buffer with 0.05% Tween-20. The KD values were calculated with the kinetics analysis (1:1 model) of Biacore T200 evaluation software. The interaction of GPR54 CT, peptides of PR motifs with Src or DUSP18, and the interaction of DUSP18 and Src were analyzed respectively by regeneration with pH 2.0 Gly-HCl buffer.

### Protein expression and purification

The C terminus of human GPR54 (H329- L398) and mouse Gpr54 (R331- L396) cDNAs were subcloned into a pGEX-4T2 vector. For purification of full-length WT human DUSP18 and DUSP18 (C104S) proteins, DUSP18 was cloned into pMCSG7 vector. For purification of Src (G85-L536) protein, cDNAs of human Src were subcloned into pFastBac HT B vector with an N-terminal 6xHis tag and expressed in Sf9 cells (Expression Systems) using the Bac-to-Bac Baculovirus Expression System (Invitrogen) for 48 h. For purification of the Src (G85-V247)-GPR54 (333–356 with C338S, C340S mutation) recombinant fusion protein, the C-terminus of GPR54 (333–356 with C338S and C340S mutation) was linked to the C terminus of Src SH3-SH2 domain (G85-V247), which was subcloned into the pMCSG7 vector.

### Protein crystallization and structure determination

For crystallization, the C-terminus of GPR54 (333–356 with mutations C338S and C340S) was inserted into the C-terminus of Src SH3-SH2 domains (G85-V247), this fragment (Src^G85-V247^–GPR54^333–356^) was then subcloned into pMCSG7. The concentrated fusion protein was set up for crystallization using a hanging drop with NT8 (Formulatrix). Diffraction data for the chimera protein (Src^G85-V247^–GPR54^333–356^) were collected at a wavelength of 0.979 Å at beamline BL17U1 at SSRF, and indexed, integrated, and scaled using the automatic XIA2 software package^48^. The structure was solved by the molecular replacement method using SH3 and SH2 domains of Src as a searching model. Refinement was carried out using Phenix^49^ and with manual adjustments with Coot^50^. Refinement parameters were summarized in Supplementary Table 4.

### Immunoblots (IB)

Whole-cell proteins were extracted using 1x RIPA buffer (25 mM Tris, pH7.5, 150 mM NaCl, 0.5% Sodium deoxycholate, 1% Triton-X-100) supplemented with protease inhibitors (Complete Mini, Roche) and phosphatase inhibitors (phosphatase inhibitor cocktail set I and II, Calbiochem). Immunoprecipitation and immunoblotting with indicated antibodies were performed as described in Supplementary Table 1. Image Jsoftware (V1.52) was used for quantification analysis. Uncropped and unprocessed blots are presented in the Supplementary Information file.

### Osteoclast differentiation

For osteoclast differentiation analyses in vitro, we isolated BMMs from femurs and tibias of 8-week-old WT and *Kiss1*^−/−^, *Gpr54*^−/−^ and *Dusp18*^−/−^ mice using a 1 ml syringe with a 26 G needle. The differentiation experiments were conducted in triplicate. BMMs were seeded into 48-well plates at a concentration of 1 × 10^4^ cells per well. Cells were incubated with 50 ng/ml RANKL (R&D, 462-TEC) and 10 ng/ml M-CSF (R&D, 416-ML), and the differentiation medium was changed every other day for 5–7 days. Osteoclasts were fixed and stained using the TRAP staining kit (Sigma-Aldrich, 387A-1KT).

### Actin-ring formation assay and pit assay

BMMs (5 × 10^3^ cells/well) were seeded into 96-well plates with a bone slice and incubated with M-CSF (10 ng/ml) and RANKL (50 ng/ml) with or without Kp-10, and the culture medium was changed every other day for 5–7 days. Osteoclasts were fixed and stained by phalloidin and DAPI. “Pits” on bone slice after osteoclast resorption were fixed with 4% Glutaraldehyde and stained with 1% toluidine blue, and pit depth was examined by laser-scanning confocal microscopy. For primary cultures of giant-cell tumor of bone (GCTB) cells, the use of all patient-derived tumor specimens was approved by the Institutional Review Board and the research ethics committee of Shanghai Changzheng Hospital, which appeared in the proceedings of the meeting of the Ethics Committee on 18 November 2014 (report number: 2014090). Informed consent obtained from all tissue donors was written. The GCTB cells were isolated from tumor samples derived from tumor resections in Shanghai Changzheng Hospital, 1 × 10^6^ GCTB cells were seeded in a 24-well plate. Cells were stimulated with indicated doses of Kp-10 for 5–7 days. The medium was changed every 2 days. Osteoclasts were fixed and stained using the TRAP-staining kit (Sigma-Aldrich, 387A-1KT).

### Osteoblast differentiation

For osteoblast differentiation analyses in vitro, we isolated bone marrow mesenchymal stem cells (MSCs) from femurs and tibias of 8-week-old WT and *Kiss1*^−/−^, *Gpr54*^−/−^ and *Dusp18*^−/−^ mice using a 1 ml syringe with a 26 G needle. MSCs were seeded into 12-well plates at a concentration of 1 × 10^5^ cells per well. Cells were incubated with 50 µg/mL ascorbic acid, 10 mmol/L beta-glycerophosphate and 10 nmol/L dexamethasone. The medium was replaced every 2 days during the incubation period. Alkaline phosphatase (ALP) activity was assayed using the assay mixtures containing 0.1 M 2-amino-2-methyl-1-propanol, 1 mM MgCl2, 8 mM p-nitrophenylphosphate disodium (Sigma). For determination of mineralization, cells were fixed with 95% ethanol and stained with AgNO3 by the von Kossa method.

### In vitro phosphatase activity assay

For the total phosphatase activity assay, the Co-IP experiment was carried out after Kp-10 treatment of RAW264.7 cells for 20 min, followed by lysis and incubation with the Gpr54 antibody. The assay was performed according to the phosphatase assay kit (Sigma-Aldrich,17-313). For the DUSP18 phosphatase activity assay, His-DUSP18 and His-DUSP18 (C104S) were purified from *E Coli*, and His-Src was purified from Sf9 insect cells. His-DUSP18 and His-DUSP18 (C104S) were incubated with His-Src in 1x phosphatase buffer (20 mM HEPES, 20 mM MgCl2, 0.03% β-mercaptoethanol)^51^ for 30 min at 30 °C with gentle shaking. The reactions were stopped by the addition of 3× SDS sample buffer followed by boiling for 10 min and subjected to Western blotting using specific antibodies.

### Mass spectrometry analyses

For mass spectrometry (MS) analysis, anti-Gpr54 IP was performed with the whole-cell lysates derived from two 10-cm dishes of RAW264.7 cells with or without Kp-10 treatment for 20 min. The protein complexes after Co-IP were extensively washed with PBS, followed by on-bead digestion. Mass spectra were acquired on a Q-Exactive mass spectrometer (Thermo Scientific, Bremen, Germany). The raw mass spectrometry data were searched against the mouse IPI databases (version 3.86, released on June 28, 2012) using the Proteome Discoverer software suite (Thermo Scientific, San Jose, USA) utilizing a label-free quantification feature. The mass spectrometry proteomics data have been deposited to the ProteomeXchange Consortium via the PRIDE^52^ partner repository with the dataset identifier PXD038433.

### Immunofluorescence (IF) staining

For endogenous IF staining, BMMs were seeded on confocal dishes and induced by 50 ng/ml RANKL and 10 ng/ml M-CSF for 2 days, and subsequently incubated with or without Kp-10 for 20 min. Cells were fixed in 10% TCA and incubated with anti-Gpr54 (Cell Signaling Technologies, 13776, 1:100), anti-Dusp18 (Santa Cruz., sc-376923, 1:100), or anti-Src (Cell Signaling Technologies, 2110, 1:400) or anti-Src (Cell Signaling Technologies, 2109, 1:400) overnight. IF staining of membrane colocation of Gpr54 with Src or Dusp18, and Src with Dusp18 were detected by Alexa Fluor™ 594 Tyramide SuperBoost™ Kit (Invitrogen, B40925) and Alexa Fluor™ 488 Tyramide SuperBoost™ Kit (Invitrogen, B40912) according to manufacturer’s instructions. The images were obtained by total internal reflection fluorescence (TIRF) microscopy (Ti2-E + H-TIRF, Nikon).

### Knockdown of gene expression by shRNA and siRNA

Lentiviral shRNA plasmids and siRNA oligos were ordered from GENEWIZ (Suzhou, China). The shRNA sequence against mouse Scr is 5′- AGCCGCCAATATCCTAGTA −3′. The siRNA sequence against mouse ERK1/2, human ERK1/2, and human Src were listed in Supplementary Table 2.

### Quantitative PCR analysis

Total RNA was extracted from RAW264.7 cells using TRIzol® (TaKaRa) and then reverse transcribed into cDNA using the PrimeScript RT-PCR Kit (TaKaRa, RR014) according to the manufacturer’s instructions. The cDNA products were used for quantitative PCR analysis by SYBR Mix (TaKaRa, RR064). Sequences of the indicated primers are described in Supplementary Table 2.

### Kisspeptins Elisa assay

Conditional medium was obtained from the process of osteoclast and osteoblast differentiation. The same number of MSC or BMMs were seeded on every 10-cm dishe to induce osteoclast differentiation. Serum was from Sham-operated and ovariectomized mice two months after operation. Level of Kisspeptins was measured using a mouse Kisspeptin ELISA kit (LS-F11879-1, LSBio).

### Micro-CT analyses

3D micro-CT analyses were performed as previously described^53^. We scanned the femur using in vitro X-ray microtomography (Skyscan 1272, Bruker micro CT) at a pixel size of 9 µm, and analyzed the results according to the manufacturer’s instructions. Region-of-interest (ROI) was defined from 10 to 110 image slices, where the growth plate slice was defined as 0 mm. The contrast was defined from 68–255; 3D analysis, BMD, and 3D models were analyzed using CTAn software (Bruker micro CT). 3D models were adjusted in CT Vox software (Bruker micro CT).

### Mice

Generation of *Gpr54* mice (strain C57/BL/6) was obtained from Dr. Eric L. Gustafson at Schering-Plow Research Institute (Kenilworth, NJ, USA). Genotyping was conducted by PCR as previously described^54^. Briefly, mutant mice (*Gpr54*^−/−^) with a targeted disruption of a 52 bp fragment from exon 2 of the GPR54 gene and a replacement with an IRES-LacZ Neo insert were generated by Deltagen (Palo Alto, CA)^54^. Generation of *Gpr54*^flox/flox^ mice (strain C57/BL/6) and *Kiss1*^flox/flox^ mice (strain C57/BL/6) were performed using the CRISPR/Cas9 system in the C57BL/6 J mouse strain from the Animal Center of East China Normal University. Edited alleles are generated by Cas9-mediated homologous recombination. Loxp sequences are inserted on each side flanking exon 2 of Gpr54 (left: 79755124–79755125, right: 79755550–79755551), and exon 2 of Kiss1 (left: 133256970–133256971, right: 133257493–133257494), which were described in Supplementary Fig. 10a (*Gpr54* floxed mice) and Supplementary Fig. 10b (*Kiss1* floxed mice). Generation of *Kiss1*^−/−^ mice was performed using the CRISPR/Cas9 system in the C57BL/6 J mouse strain from the Animal Center of East China Normal University. A sgRNA targeting CCTGGATCCACAGGTACGCAC of the *Kiss1* gene was designed and 13 bp (CCTGGATCCACAG) of the *Kiss1* gene was deleted. Genotyping was performed by PCR as described in Supplementary Table 2. Generation of *Dusp18*^−/−^ mice was performed using the CRISPR/Cas9 system in the C57BL/6 J mouse strain from the Animal Center of East China Normal University. Two 20-bp sgRNAs targeting TGCGAGAGGCCTCTGATCGAAGG and GCGACGGGCGCATCGACCACAGG were designed and 164 bp between 406 bp and 569 bp of the Dusp18 gene was deleted. Genotyping was performed by PCR as described in Supplementary Table 2. Generation of *Arrb1*^−/−^ and *Arrb2*^−/−^ mice (strain C57/BL/6) were described in a previous publication^55,56^. G_q_α^flox/flox^:G_11_α^−/−^ mice (strain C57/BL/6) wre obtained from Dr. Richard D Ye, at The Chinese University of Hong Kong (Shenzhen, China), in which the gene coding for Gαq, gnaq, is flanked with loxP sites^57^ were crossed to the constitutively Gα11^−/−^ mice^58^, and genotyping was conducted by PCR as previously described^57,58^. LysM-Cre mice (strain C57BL/6) were described in ref. ^59^. *Gpr54*^flox/flox^ mice, *Kiss1*^flox/flox^ mice and Gqα^flox/flox^:G11α^−/−^ mice were crossed to LysM-Cre mice respectively to abtain osteoclast conditional knockout of *Gpr54, Kiss1* or *G*α*q* mice. Both male and female mice were used in all experiments, except for the OVX model that only used female mice. All of the mice were randomly assigned to groups. Animal study was approved by the Institutional Animal Care and Use Committee at East China Normal University (m20140907). Mice were maintained, bred and studied in compliance with approved protocols. Mouse euthanasia was done first by anesthesia and then followed by cervical dislocation. Animal experiments were conducted in accordance with the ARRIVE guidelines.

### Histology

Bone tissue was removed of excess tissues and fixed with 4% formalin, decalcified with 0.5 M EDTA for 10–14 days, embedded with paraffin, sectioned, and stained using the TRAP staining kit (Sigma-Aldrich, 387A-1KT). The third lumbar spine vertebrae (L3) were fixed with 4% formalin, sectioned, and subjected to Von Kossa staining as described previously^60,61^. For double calcian labeling assay, 4−5 week-old mice were administrated calcian (30 μg/mL) through intraperitoneal injection for the first time and the second time after 10 days. The third lumbar spine vertebrae (L3) were fixed with 4% formalin, sectioned, and subjected to Goldner’s Masson trichrome staining, or acquisition of fluorescence images. Histomorphometric measurements were made using the OsteoMeasure Analysis System (Osteometrics, Atlanta, GA, USA) according to standard criteria.

### Treatment with Kp-10 and bone targeting Kp-10 ((DSS)*6-Kp-10) in vivo

For animal studies in vivo, mice were randomized for weight. For the OVX-induced bone loss model, 2-month-old female C57BL/6 mice were sham-operated (sham) or ovariectomized (OVX) and randomly divided into indicated groups. Vehicle, Kp-10 or (DSS)*6-Kp-10 was injected into the tail vein twice per week. After two months of treatment, the femurs and the L3 lumbrae were isolated for micro-CT or histomorphometric analysis. 3D micro-CT analyses were performed according to a standard protocol. BMD and bone volume were analyzed by CT-analysis software (CTAn, Bruker micro CT, Kontich, Belgium) and images were reconstituted by CT-volume software (CTvol, skyscan, CTAn, Bruker micro CT, Kontich, Belgium).

### Statistical analyses

Data are represented as the mean ± standard error of the mean (SEM) or the mean ± standard deviation (SD) as indicated in the figure legends. The statistical significance of differential values between experiments and controls was calculated by GraphPad Prism V7. The statistical *P* values were determined by Student’s *t*-test or one-way ANOVA analysis as indicated in the manuscript.

### Reporting summary

Further information on research design is available in the Nature Portfolio Reporting Summary linked to this article.

## Supplementary information

Supplementary Information

Reporting Summary

## Source data

Source Data

## Data Availability

All relevant data are available in the main figures, supplementary information, and the source data file. Source data and full-length Western Blots are provided in the Source Data File. Mass spectrometry analysis data are available via ProteomeXchange with identifier PXD038433. Source data are provided with this paper.
