# Author Correction: Kisspeptin-10 binding to Gpr54 in osteoclasts prevents bone loss by activating Dusp18-mediated dephosphorylation of Src

**DOI:** 10.1038/s41467-024-55352-1

**Published:** 2024-12-19

**Authors:** Zhenxi Li, Xinghai Yang, Ruifeng Fu, Zhipeng Wu, Shengzhao Xu, Jian Jiao, Ming Qian, Long Zhang, Chunbiao Wu, Tianying Xie, Jiqiang Yao, Zhixiang Wu, Wenjun Li, Guoli Ma, Yu You, Yihua Chen, Han-kun Zhang, Yiyun Cheng, Xiaolong Tang, Pengfei Wu, Gewei Lian, Haifeng Wei, Jian Zhao, Jianrong Xu, Lianzhong Ai, Stefan Siwko, Yue Wang, Jin Ding, Gaojie Song, Jian Luo, Mingyao Liu, Jianru Xiao

**Affiliations:** 1 Institute of Orthopedic Biomedical and Device Innovation, School of Health Science and Engineering, University of Shanghai for Science and Technology, Shanghai, 200093 China; 2 Institute of Orthopedics, Department of Orthopedic Oncology, Shanghai Changzheng Hospital, Naval Medical University, Shanghai, 200003 China; 3 East China Normal University and Shanghai Changzheng Hospital Joint Research Center for Orthopedic Oncology, Shanghai Key Laboratory of Regulatory Biology, Institute of Biomedical Sciences and School of Life Sciences, East China Normal University, Shanghai, 200241 China; 4 Department of Pathology, Beth Israel Deaconess Medical Center, Harvard Medical School, Boston, MA 02215 USA; 5 School of Biomedical Sciences, Hunan University, Changsha, 410082 China; 6 Department of Neurology, Beth Israel Deaconess Medical Center, Harvard Medical School, Boston, MA 02215 USA; 7 Academy of Integrative Medicine, Shanghai University of Traditional Chinese Medicine, Shanghai, 201203 China; 8 Department of Translational Medical Sciences, Institute of Biosciences and Technology, Texas A&M University Health Science Center, Houston, TX USA; 9 Shanghai Key Lab of Cell Engineering; Translational Medicine Research Center, Naval Medical University, Shanghai, 200433 China; 10 Clinical Cancer Institute, Center for Translational Medicine, Naval Medical University, Shanghai, 200433 China; 11 Yangzhi Rehabilitation Hospital (Shanghai Sunshine Rehabilitation Center), Tongji University School of Medicine, Shanghai, China

Correction to: *Nature Communications* 10.1038/s41467-024-44852-9, published online 12 February 2024

In the original version of this article, Gaojie Song, Jian Luo, Mingyao Liu and Jianru Xiao were mistakenly listed as contributing authors, while they should have been listed as corresponding authors alongside Zhenxi Li, who was noted as sole corresponding author.

This error has now been corrected in the HTML and PDF versions of the Article.

