# Supplementary material for: Kisspeptin-10 binding to Gpr54 in osteoclasts prevents bone loss by activating Dusp18-mediated dephosphorylation of Src
Source: Nat Commun. 2024 Feb 12;15:1300. doi: 10.1038/s41467-024-44852-9 (PMC10861593; doi:10.1038/s41467-024-44852-9)
Supplement: Supplementary file 1 — Supplementary Information [file 41467_2024_44852_MOESM1_ESM.docx]

**Supplementary Information**

This file contains Supplementary Figures 1 to 16, and Supplementary Tables 1 to 4.

**Kisspeptins/Gpr54 Protects Bone Mass through Src Dephosphorylation by Dusp18 in osteoclasts**

Zhenxi Li^1, 2, 3, 4^ **✉**, Xinghai Yang^2^, Ruifeng Fu^1, 2^, Zhipeng Wu^2^, Shengzhao Xu^3^, Jian Jiao^2^, Ming Qian^2^, Long Zhang^1^, Chunbiao Wu^1, 2^, Tianying Xie^1, 2^, Jiqiang Yao^2^, Zhixiang Wu^2^, Wenjun Li^3^, Guoli Ma^3^, Yu You^3^, Yihua Chen^3^, Han-kun Zhang^3^, Yiyun Cheng^3^, Xiaolong Tang^5^, Pengfei Wu^6^, Gewei Lian^6^, Haifeng Wei^2^, Jian Zhao^2^, Lianzhong Ai^1^, Jianrong Xu^7^, Stefan Siwko^8^, Yue Wang^9^, Jin Ding^10^, Gaojie Song^3^, Jian Luo^11^, Mingyao Liu^3^ and Jianru Xiao^1, 2, 3^

^1^ Institute of Orthopedic Biomedical and Device Innovation, School of Health Science and Engineering, University of Shanghai for Science and Technology, Shanghai 200093, China

^2^Institute of Orthopedics, Department of Orthopedic Oncology, Shanghai Changzheng Hospital, Naval Medical University, Shanghai 200003, China

^3^East China Normal University and Shanghai Changzheng Hospital Joint Research Center for Orthopedic Oncology, Shanghai Key Laboratory of Regulatory Biology, Institute of Biomedical Sciences and School of Life Sciences, East China Normal University, Shanghai 200241, China

^4^Department of Pathology, Beth Israel Deaconess Medical Center, Harvard Medical School, Boston, MA 02215, USA

^5^School of Biomedical Sciences, Hunan University, Changsha 410082, China

^6^Department of Neurology, Beth Israel Deaconess Medical Center, Harvard Medical School, Boston, MA 02215, USA

^7^Academy of Integrative Medicine, Shanghai University of Traditional Chinese Medicine, Shanghai 201203, China

^8^Department of Translational Medical Sciences, Institute of Biosciences and Technology, Texas A&M University Health Science Center, Houston, TX, USA

^9^Shanghai Key Lab of Cell Engineering; Translational Medicine Research Center, Naval Medical University, Shanghai, 200433，China

^10^Clinical Cancer Institute, Center for Translational Medicine, Naval Medical University, Shanghai 200433, China

^11^Yangzhi Rehabilitation Hospital (Shanghai Sunshine Rehabilitation Center), Tongji University School of Medicine, Shanghai, China

These authors contributed equally to this work: Zhenxi Li, Xinghai Yang, Ruifeng Fu, Zhipeng Wu

These authors jointly supervised this work: Gaojie Song, Jian Luo, Mingyao Liu, Jianru Xiao and Zhenxi Li

*Correspondence should be addressed to Dr Zhenxi Li (zxli@usst.edu.cn).

**Supplementary Fig. 1** **a, b** IB analysis of whole cell lysates (WCL) derived from BMMs isolated from eight-week-old wild-type (WT) and *Gpr54 ^-/-^* mice. BMMs were starved in serum-free α-MEM for 4 hours and then treated with indicated doses of Kp-10 for 1 hour and followed by the addition of 50 ng/mL M-CSF, 100 ng/mL RANKL for another 30 minutes (a) and quantification of protein levels (b). **c, d** Elisa assay showing Kisspeptins derived from the process of osteoblast or osteoclast differentiation in vitro. Kisspeptins normalization was based on the same number of MSCs, BMMs which were seeded on the plates before differentiation stimulation (c), and serum Kisspeptins from Sham (n=8) and OVX (n=7) mice (d). Representative results were obtained from at least three independent experiments.  Error bars are ± SEM. *P*-values were determined by one-way ANOVA analysis (a) or two-tailed Student’s *t*-test (b). **P* <0.05, ****P* <0.001, *****P* <0.0001. Representative results were obtained from at least three independent experiments. Source data are provided as a Source Data file.

**Supplementary Fig.2 a, b** Representative images of pits formed by osteoclast derived from NC, GPR54 virus-infected BMMs. Bone resorption promoted by *Gpr54* deletion was rescued by ectopic expression of Gpr54 virus*.* **c-h** Representative images of TRAP staining. **c, d** TRAP assay showing osteoclast-like cells from GCT were dose-dependently suppressed by Kp-10. **e, f** TRAP assay showing osteoclast formation were promoted by treatment of the Gpr54 antagonist WB599 which was incubated with RANKL and M-CSF. **g, h** Bone resorption promoted by *Kiss1* deletion was rescued by ectopic expression of Kiss1*.* Error bars are ± SEM. **i, j** Representative images of pits formed by osteoclast derived from NC, or Kiss1 virus-infected BMMs. Bone resorption promoted by *Kiss1* deletion was rescued by ectopic expression of *Kiss1* virus*. P*-values were determined by one-way ANOVA analysis (b, d, f, j) or two-tailed Student’s *t*-test (h). **P* <0.05, ****P* <0.001, *****P* <0.0001. Representative results were observed from at least three independent experiments. Source data are provided as a Source Data file.


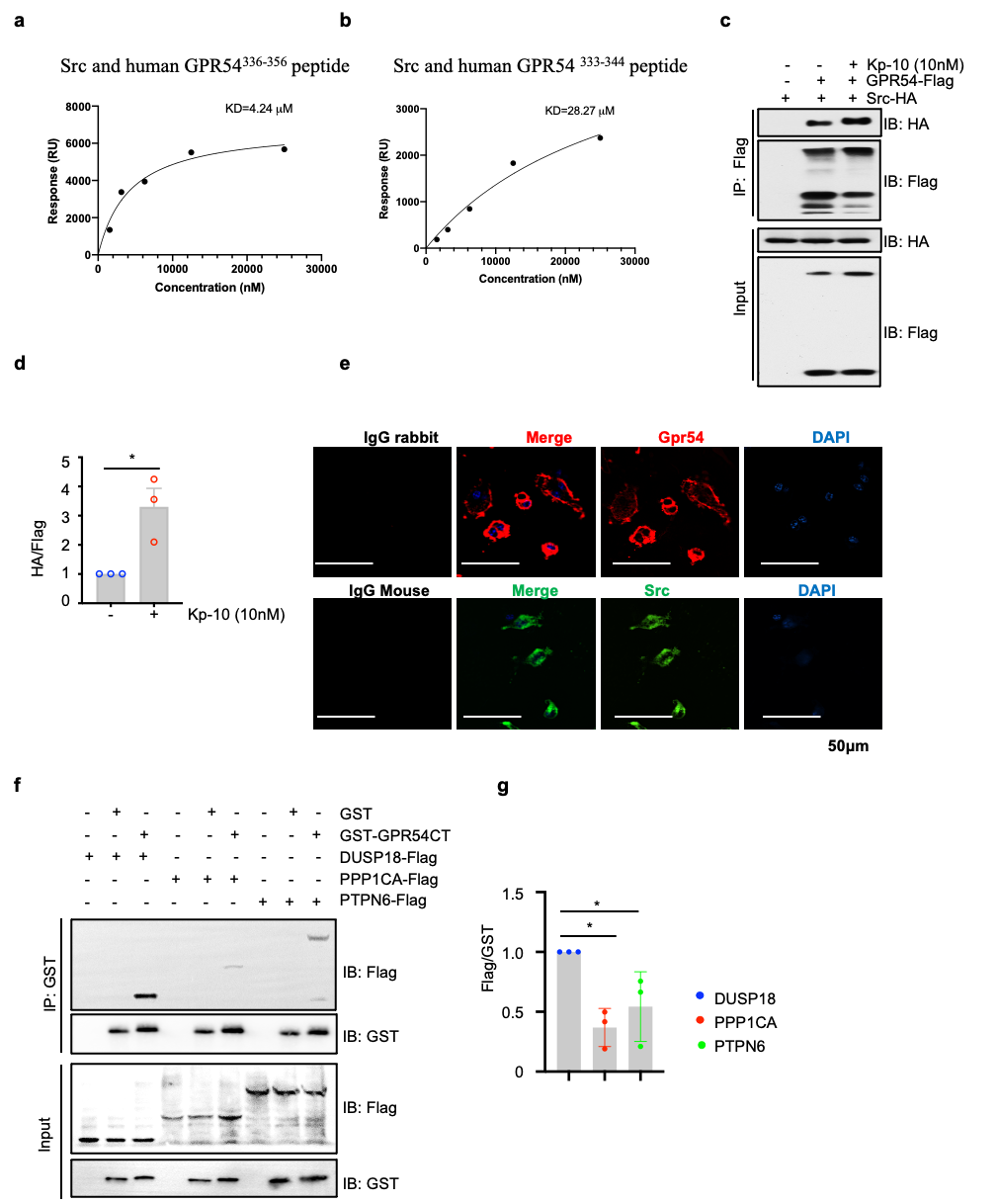


**Supplementary Fig. 3 a, b** SPR binding analysis showing the binding affinity of Src and human GPR54^336-356^ peptide (a), Src and human GPR54 ^333-344^ peptide (b). **c, d** Anti-Flag IP derived from 293T cells transfected with HA-Src and GPR54-Flag constructs with Kp-10 (10 nM) stimulation (c) and quantification of protein levels (d). **e.** **e** IF staining was carried out using pre-osteoclasts differentiated from BMMs in the presence of M-CSF (10 ng/ml) and RANKL (50 ng/ml) for 2 days. Representative images were shown by TIRF microscopy. **f, g** IB analysis of total samples and GST pull-downs using GST proteins including GST, GST-GPR54 CT incubated separately with WCL of 293T cells transfected with DUSP18-flag, PPP1CA-Flag or PTPN6-Flag (f), and quantification of protein levels (g). Error bars are ± SEM. *P*-values were determined by two-tailed Student’s *t*-test (d) or one-way ANOVA analysis (g). **P* <0.05, Representative results were observed from at least three independent experiments. Source data are provided as a Source Data file.

Supplementary Fig. 4 **a, b** Immunoblots showing Kiss1, Gpr54, and Dusp18 expression during osteoclast differentiation (a) and quantification of protein levels (b). **c** Representative images of IHC staining showing Kiss1, Gpr54 and Dusp18 expression in the femurs of Sham-operated mice and ovariectomy (OVX) mice (n=3 per group), tissues of giant cell tumor of bone (GCTB) (n=10, 4 males and 6 females). Error bars are ± SEM. *P*-values were determined by one-way ANOVA analysis (b). **P* <0.05, ***P* <0.01, *****P* <0.0001. Source data are provided as a Source Data file.


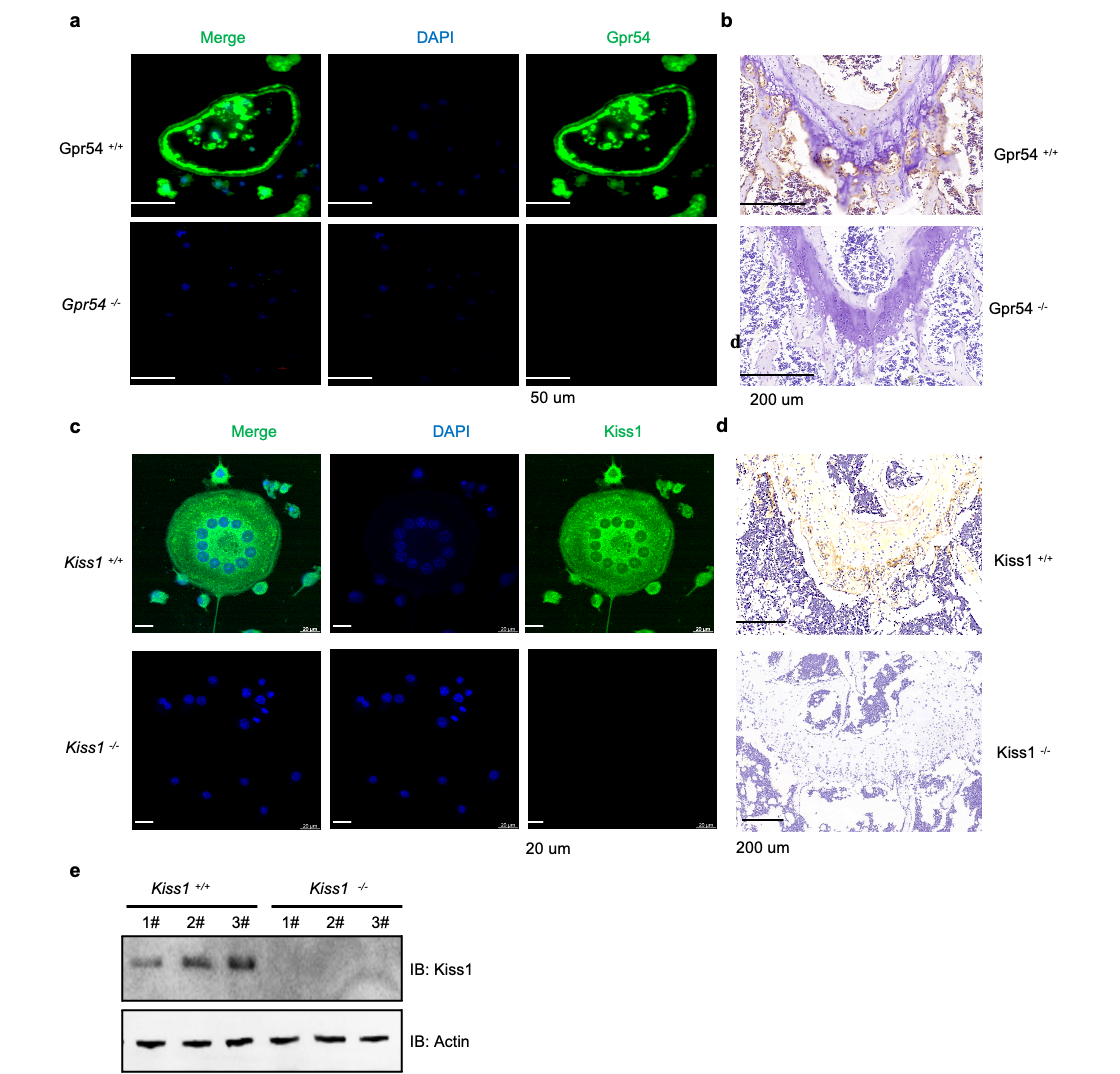


Supplementary Fig. 5 **a, c** IF staining showing the signal of Gpr54 in the osteoclast in vitro derived from Gpr54 ^+/+^ and Gpr54 ^-/-^ mice (a) and Kiss1 in the osteoclast derived from Kiss1^+/+^ and Kiss1 ^-/-^ mice (c), n=3 per group. **b, d** Representative images of IHC staining showing Gpr54 expression in the femurs of Gpr54 ^+/+^ and Gpr54 ^-/-^ mice (c), and or Kiss1 expression in the femurs of Kiss1^+/+^ and Kiss1 ^-/-^ mice (d), n=3 per group. **e** Immunoblots showing the expression of Kiss1 in wild-type and Kiss1 knockout BMMs.

**
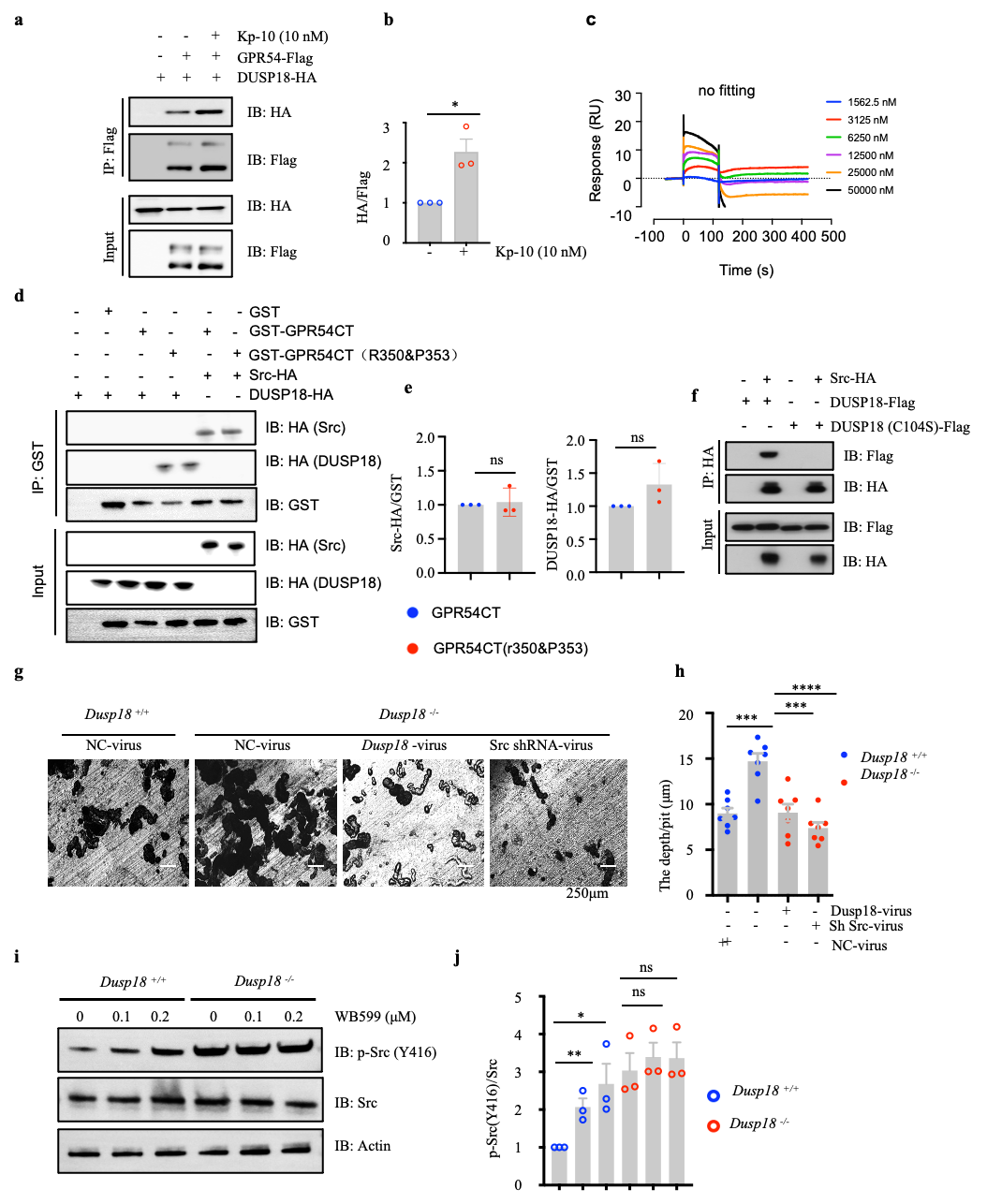
**

**Supplementary Fig. 6** **a, b** IB and quantification of protein levels analysis. Anti-Flag IP derived from 293T cells transfected with DUSP18-HA, GPR54-Flag constructs with Kp-10 (10 nM) stimulation for 20 minutes (a) and quantification of protein levels (b). **c** SPR binding analysis showing the unfitting binding affinity of Dusp18 and mouse Gpr54 ^339-344^ peptide (c). **d-f** IB and quantification of protein levels analysis. GST pull-downs using GST proteins including GST, GST-GPR54CT, GST-GPR54CT (R350A&P353A) incubated with WCL of 293T cells transfected with DUSP18-HA or SRC-HA (d) and quantification of protein levels (e). **f** Anti-Flag IP derived from 293T cells transfected with Src-HA, DUSP18-Flag, DUSP18 (C104S)-Flag constructs. **g, h** Representative images of pits assay showing osteoclast resorption enhanced by *Dusp18* deletion was rescued by ectopic expression of Dusp18 or knockdown of Src by shRNA. **i, j** IB and quantification of protein levels analysis. Phosphorylation of Src was enhanced in WT BMMs but not in *Dusp18* ^-/-^ BMMs by treatment of WB599 overnight (i) and quantification of protein levels (j). Error bars are ± SEM. *P*-values were determined by two-tailed Student’s *t*-test (b, e) or by one-way ANOVA analysis (h, j). **P* <0.05, ***P* <0.01, ****P* <0.001, *****P* <0.0001. Representative results were observed from at least three independent experiments. Source data are provided as a Source Data file.

**Supplementary Fig. 7 a-b** IB and quantification of Dusp18 protein level induced by indicated dose of Kp-10 for 1 hour in RAW264.7 cells (a) and quantification of protein levels (b) or induced by Kp-10 (1nM) for 1 hour in RAW264.7 transfected with ERK1/2 siRNA. Error bars are ± SEM. *P*-values were determined by one-way ANOVA analysis (b). *****P* <0.0001. “ns” means no significant difference. Representative results were observed from at least three independent experiments. Source data are provided as a Source Data file.

**Supplementary Fig. 8** **a-d** IB and quantification of protein levels. Anti-Flag IP derived from 293T cells transfected with DUSP18-HA, GPR54-Flag and Src siRNA (a) and quantification of protein levels (b) or ERK siRNA (c, d) with Kp-10 (10 nM) stimulation for 20 minutes (c) and quantification of protein levels (d). Error bars are± SEM. *P*-values were determined by one-way ANOVA analysis (b, d, f, h). **P* <0.05, “ns” means no significant difference. Representative results were observed from at least three independent experiments. Source data are provided as a Source Data file.


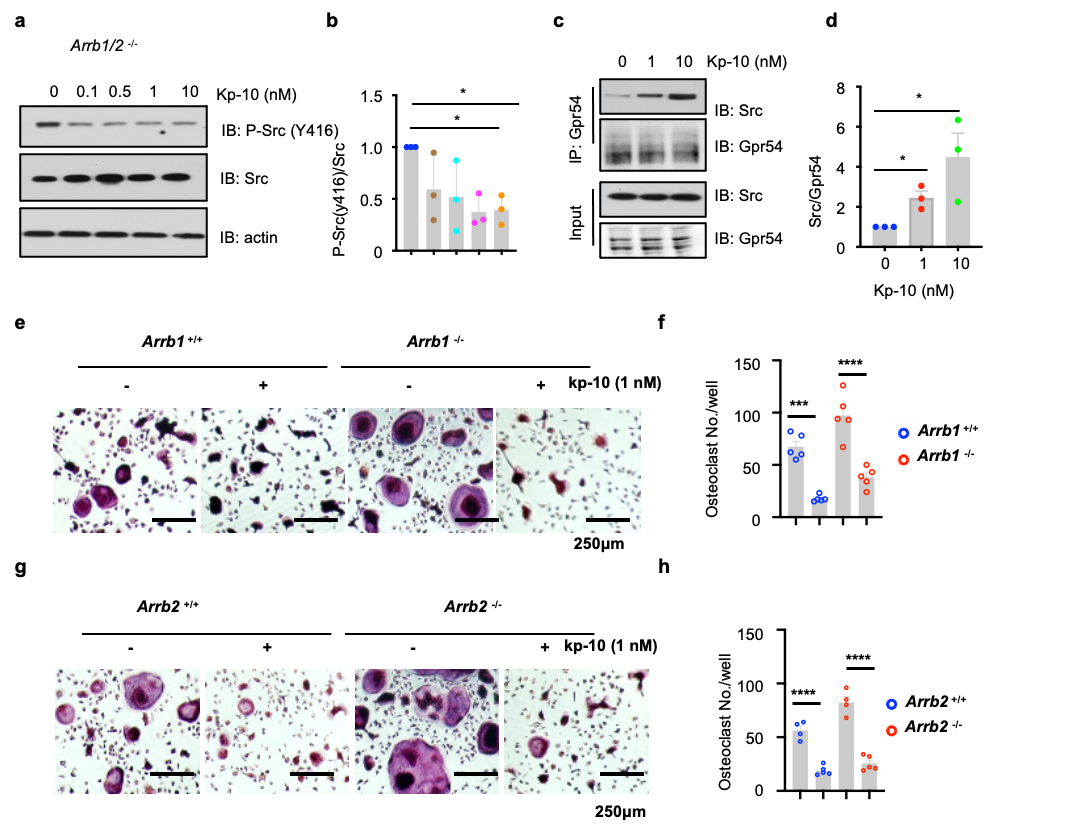


**Supplementary Fig. 9 a-d** IB and quantification of protein levels analysis. Phosphorylation of Src was still suppressed upon Kp-10 stimulation for 1 hour in *Arrb1* and *Arrb2* double knockout MEFs (a) and quantification of protein levels (b). Anti-Gpr54 IP derived from Raw264.7 cells with indicated dose of Kp-10 stimulation for 20 minutes (c) and quantification of protein levels (d). Representative images of TRAP staining assay showing osteoclast formation was still suppressed by Kp-10 both in *Arrb1* KO (e, f) or *Arrb2* KO (g, h) BMMs. Error bars are± SEM. *P*-values were determined by two-way ANOVA analysis (b, d, f, h) **P* <0.05, *****P* <0.0001. “ns” means no significant difference. Representative results were observed from at least three independent experiments. Source data are provided as a Source Data file.

**Supplementary Fig. 10** **a** Targeting strategy of *Gpr54.* Genomic structure of the WT mouse *Gpr54* gene. Exon 2 was flanked by Loxp sequences. The modified *Gpr54* locus after homologous recombination and the deleted *Gpr54* gene after Cre-mediated excision of exon 2 is shown. **b** Targeting strategy of *Kiss1.* Genomic structure of the WT mouse *Kiss1* gene. Exon 2 was flanked by Loxp sequences. The modified *Kiss1* locus after homologous recombination and the deleted *Kiss1* gene after Cre-mediated excision of Exon 2 is shown. **c-f,** Elisa assay showing testosterone derived from the serum of male *Gpr54^f/f^, Gpr54cko* mic (c), LH and FSH derived from the serum of female *Gpr54^f/f^ and Gpr54cko mice* (d), testosterone derived from the serum of male *Kiss1^f/f^, Kiss1cko mice* (e), LH and FSH derived from the serum of female *Kiss1^f/f^, Kiss1cko mice* (f), n=6 per group. Error bars are ± SEM. *P*-values were determined by unpaired two-tailed Student’s t-test (c-f). “ns” means no significant difference. Source data are provided as a Source Data file.


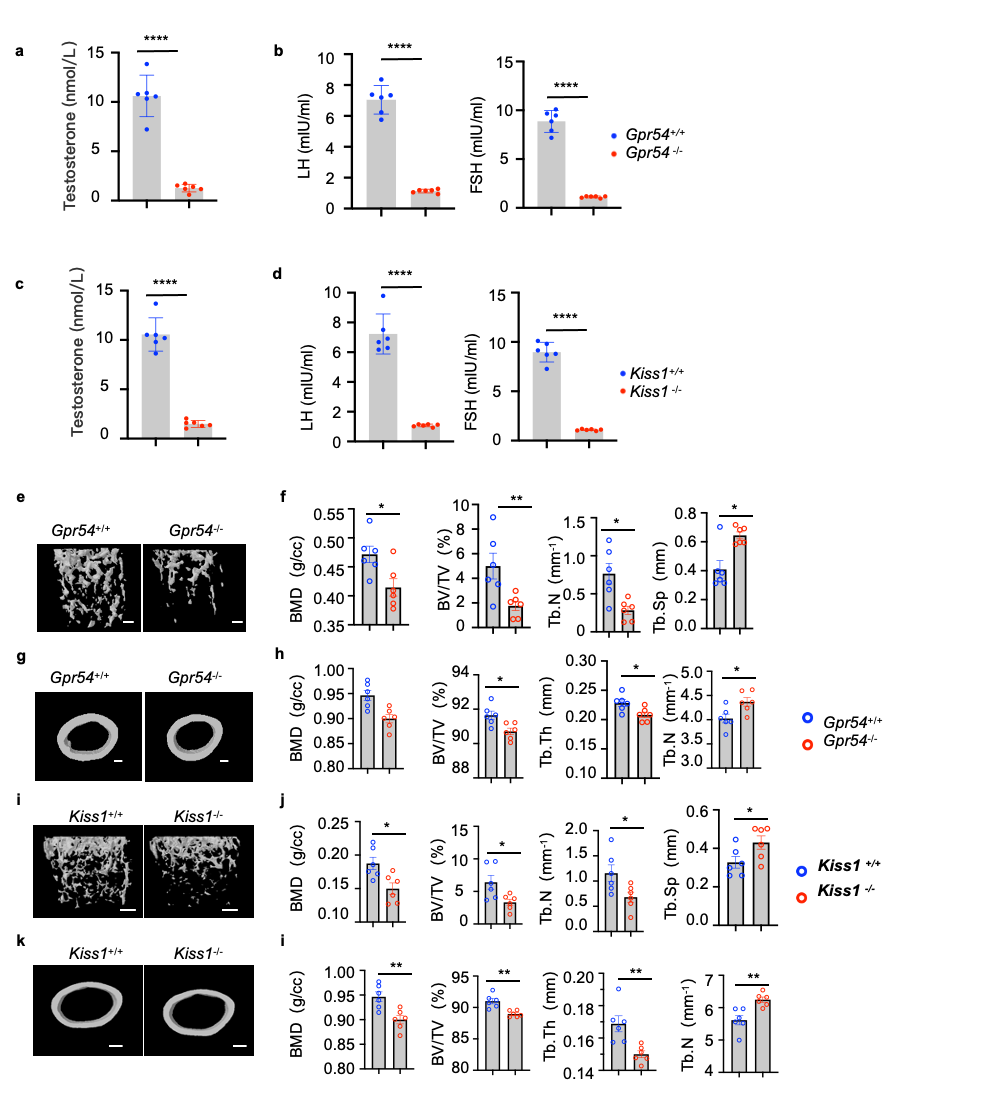


**Supplementary Fig. 11** **a-d** Elisa assay showing testosterone from the serum of male *Gpr54 ^+/+^, Gpr54 ^-/-^ mice* (a), LH and FSH derived from the serum of female *Gpr54 ^+/+^, Gpr54 ^-/-^ mice* (b), testosterone from the serum of male *Kiss1 ^+/+^, Kiss1 ^-/-^ mice* (c), LH and FSH derived from the serum of female *Kiss1 ^+/+^, Kiss1 ^-/-^ mice* (d), n=6 per group. **e, g, i, k** Representative micro-CT images. 8-week-old WT and *Gpr54 ^-/-^* mice (a, c), 8-week-old WT and *Kiss1 ^-/-^* mice (e, g). **f, h, g, l** Bone parameter analysis of the mice from a, c or e, g. BMD, bone mineral density; BV/TV, bone volume as a fraction of total bone volume; Tb. Th, trabecular thickness; Tb. N, trabecular number; Tb. Sp, trabecular separation. n = 6 per group including 3 female and 3 male mice. Error bars are ± SEM. **P* < 0.05, ***P* < 0.01, unpaired two-tailed Student’s t-test. Scale bar, 500 µm. Representative results were observed from at least three independent experiments. Source data are provided as a Source Data file.

**Supplementary Fig. 12** **a** Representative TRAP staining images of the whole calvaria of indicated WT, *Kiss1* ^-/-^ (n=5 per group, 3 females and 2 males) and *Gpr54* ^-/-^ mice (n=5 per group,1 female and 4 males), 8w (OVX) means that the mice were ovariectomized at one week old and performed TRAP staining assay at 8-week-old. Scale bar, 2 mm. **b** Osteoclast surface analysis, Oc. S/BS means the ratio of osteoclast surface and total calvaria surface. Error bars are± SEM. **P* < 0.05, ***P* < 0.01. unpaired two-tailed Student’s t-test. Representative results were observed from at least three independent experiments. Source data are provided as a Source Data file.

**Supplementary Fig. 13 a, c, e, g** Representative double calcian labeling images of the third lumbar spine vertebrae from 6-week-old *Gpr54* ^-/-^ (n=6 for WT mice including 3 female and 3 male mice, n=7 for *Gpr54* ^-/-^ mice including 3 female and 4 male mice), *Kiss1* ^-/-^ (n=6 per group including 3 female and 3 male mice, *Gpr54* ^f/f^ and *Gpr54* cKO (n=4 per group including 2 female and 2 male mice), *Kiss1*^f/f^ and *Kiss1* cKO (n=4 per group including 2 female and 2 male mice). **b,** **d, f, h** Parameters of MAR (mineral apposition rate), BFR (bone formation rate)/ BS (bone surface) were analyzed. **i, k** Representative Goldner's Masson trichrome staining images from the third lumbar spine vertebrae from *Gpr54* ^-/-^, *Kiss1*^-/-^ and the comparing WT mice. **j, l** Osteoblast parameters of 6-week-old WT and *Gpr54* ^-/-^, *Kiss1*^-/-^ including osteoblast number (N. Ob/B. Pm), osteoblast surface/bone surface (Ob. S/BS), osteoid surface (OS/BS). Error bars are ± SEM. **P* < 0.05, ***P* < 0.01, unpaired two-tailed Student’s t-test. Scale bar, 100 µm. Representative results were observed from at least three independent experiments. Source data are provided as a Source Data file.

**Supplementary Fig. 14 a** Representative Goldner's Masson trichrome staining images from the third lumbar spine vertebrae from *Dusp18* ^-/-^ and the comparing WT mice. **b** Osteoblast parameters of 6-week-old WT and *Dusp18* ^-/-^ (n=6 per group including 3 female and 3 male mice) including osteoblast number (N. Ob/B. Pm), osteoblast surface/bone surface (Ob. S/BS), osteoid surface (OS/BS). Error bars are ± SEM. ***P* < 0.01, unpaired two-tailed Student’s t-test. Scale bar, 100 µm. Representative results were observed from at least three independent experiments. Source data are provided as a Source Data file.

**Supplementary Fig. 15 a, c** Representative images of ALP staining showing that osteoblast differentiation was enhanced when *Gpr54* or *Kiss1* was deleted (a), and suppressed by Kp-10 stimulation (c). **b, d** Representative images of Von Kossa staining showing the mineralization was promoted by *Gpr54* or *Kiss1* deletion (b) and inhibited by Kp-10 stimulation (c). Scale bar, 5 mm.

**Supplementary Fig. 16 a** Representative image of Von Kossa staining of sham-operated female C57 mice treated with vehicle (Sham, n=4), ovariectomized mice (OVX, n=3), and OVX mice treatment with 1 nmol/kg Kp-10 (n=6),10 nmol/kg Kp-10 (n=6),1 nmol/kg (DSS)*6-Kp-10 (n=5) or 10 nmol/kg (DSS)*6-Kp-10 (n=6) twice one week by intraperitoneal injection for two months. Scale bar, 200 µm. **b** bone parameter analysis of the samples above showing that (DSS)*6-Kp-10 rescued OVX induced bone loss more effectively than Kp-10. BV/TV, bone volume as a fraction of total bone volume; Tb. Th, trabecular thickness; Tb. Sp, trabecular separation; Tb. N, trabecular number. **c** Elisa assay showing LH, FSH derived from the serum of sham-operated female C57 mice treated with vehicle (Sham+ vehicle) or 10 nmol/kg (DSS)*6-Kp-10 (Sham+10 nmol/kg Kp-10), ovariectomized mice treated with vehicle (OVX+ vehicle) or 10 nmol/kg (DSS)*6-Kp-10 (OVX+ 10 nmol/kg Kp-10) twice one week by intraperitoneal injection for two months. n=6 per group. Error bars are ± SEM. *P*-values were determined by two-way ANOVA analysis (b, c) ****P* <0.001, *****P* <0.0001. “ns” means no significant difference. Representative results were observed from at least three independent experiments. Source data are provided as a Source Data file.

| **Supplementary Table 1 Antibodies used in this study** | | |
| --- | --- | --- |
| **Antibody** | **Source** | **Dilutions ((WB, Western blotting; IP, immunoprecipitation, IHC, IF)** |
| p-Src (Tyr416) | CST (#2101) | WB (1: 1,000) |
| Src | CST (#2109) | WB/IP/IF (1: 1,000/1:50/1:400) |
| Src | CST (#2110) | WB/IF (1: 1,000/1:400) |
| Phospho-IKKα/β (Ser176/180) | CST (#2697) | WB (1: 1,000) |
| IKKβ | CST (#8943) | WB (1: 1,000) |
| p-p38 (Thr180/Tyr182) | CST (#9211) | WB (1: 1,000) |
| p38 | CST (#9212) | WB (1: 1,000) |
| p- JNK(Thr183/Tyr185) | CST (#9251) | WB (1: 1,000) |
| JNK | CST (#9252) | WB (1: 1,000) |
| p-Erk1/2(Thr202/Tyr204) | CST (#4370) | WB (1: 1,000) |
| Erk1/2 | CST (#4695) | WB (1: 1,000) |
| KISS1R | CST (#13776) | WB/IP/IF/IHC (1:1,000/1:50/1:100/1:100) |
| KiSS-1 | Santa Cruz (sc-101246) | WB/ IHC (1: 500/1:50) |
| DUSP18 | Santa Cruz (sc-376923) | WB/IP/IF/IHC (1:500/1:20/1:100/1:100) |
| NFATc1 | Santa Cruz (sc-7294) | WB (1: 500) |
| Actin | CST (#3700) | WB (1: 1,000) |
| VINCULIN | Sigma (V4505) | WB (1: 5,000) |
| Rabbit IgG | CST (#2729) | WB (1: 1,000) |
| GST-tag | CST (#2624) | WB (1: 1,000) |
| Myc-tag | CST (#2272) | WB (1: 1,000) |
| Rabbit HA-tag | CST (#3724) | WB (1: 1,000) |
| Mouse HA-tag | Santa Cruz (sc-7392) | WB (1: 1,000) |
| Mouse Flag-tag | Sigma (F3165) | WB (1: 5,000) |
| Rabbit Flag-tag | Sigma (F7425) | WB (1: 5,000) |
| peroxidase-conjugated mouse secondary antibody | Sigma (A4416) | WB (1: 5,000) |
| peroxidase-conjugated rabbit secondary antibody | Sigma (A4914) | WB (1: 5,000) |

| Supplementary Table 2 Oligonucleotides used in this study | | |
| --- | --- | --- |
| targets | Sequence (5’-3’) | purpose |
| mERK1 | 5′-UGA CCA CAU CUG CUA CUU C-3′ | siRNA |
| mERK2 | 5′-GUG CUG UGU CUU CAA GAG C-3′ | siRNA |
| hERK1 | 5′-GCC AUG AGA GAU GUC UAC A-3′ | siRNA |
| hERK2 | 5′-GAG GAU UGA AGU AGA ACA G-3′ | siRNA |
| h Src | 5′-GGG AGA ACC UCU AGG CAC A-3′ | siRNA |
| m Src | 5′-AAGATCACTAGACGGGAATCA-3′ | shRNA |
| mDusp18F | 5′-GTC CCT TCC ATT GTT CAC GG-3′ | qRT-PCR |
| mDusp18R | 5′-GGG ACA TGG CAT GGT ACT TCA-3′ | qRT-PCR |
| mActinF | 5′-GTA CGC CAA CAC AGT GCT G-3′ | qRT-PCR |
| mActinR | 5′-CGT CAT ACT CCT GCT TGC TG-3′ | qRT-PCR |
| mKiss1F | 5′-GAT TCC GTT GCC GAC CGT AT-3′ | Genotype |
| mKiss1R | 5′-GGG ACA TGG CAT GGT ACT TCA-3′ | Genotype |
| mGpr54 WT | 5′-GCC TAA GTT TCT CTG GTG GAG GAT G-3′ | Genotype |
| mGpr54 both | 5′-CGC GTA CCT GCT GGA TGT AGT TGA C-3′ | Genotype |
| mGpr54 KO | 5′-GTG GGA TTA GAT AAA TGC CTG CTC T-3′ | Genotype |
| KISS1-L-loxp-F | 5′-TCT GGC AAG CAC TTG AAA G-3′ | Genotype |
| KISS1-L-loxp-R | 5′-ATA CCG CGA TTC CTT TTC C-3′ | Genotype |
| KISS1-R-loxp-F | 5′-CCA AGG CAG GGA GCT TCT A-3′ | Genotype |
| KISS1-R-loxp-R | 5′-CCT CAG TGG CCG AGT TTC T-3′ | Genotype |
| GPR54-L-Ioxp-F | 5′-TGT CTT ATG GAC GTG ATA GCC-3′ | Genotype |
| GPR54-L-loxp-R | 5′-GCG GAT GCT GGA AGA TGG-3′ | Genotype |
| GPR54-R-loxp-F | 5′-CCT TCA CCG CAC TCC TCT A-3′ | Genotype |
| GPR54-R-loxp-R | 5′-CAC AGA CCC TGA CCA CAA CA-3′ | Genotype |
| Gna^+^ | 5′-AGC TTA GTC TGG TGA CAG AAG C-3′ | Genotype |
| Gnaq^flox^ | 5′-GCA TGC GTG TCC TTT ATG TGA G-3′ | Genotype |
| Gna11^+^ -F | 5′-GCC CCT TGT ACA GAT GGC AG-3′ | Genotype |
| Gna11^+^ -R | 5′-AGC ATG CTG TAA GAC CGT AG-3′ | Genotype |
| Gna11^-^ -F | 5′-CAG GGG TAG GTG ATG ATT GTG C-3′ | Genotype |
| Gna11^-^ -R | 5′-GAC TAG TGA GAC GTG CTA CTT CC-3′ | Genotype |
| Dusp18 F | 5′-GTC CCT TCC ATT GTT CAC GG-3′ | Genotype |
| Dusp18 R | 5′-TCA AAC GGG TCT CCT TCT CG-3′ | Genotype |
| Arrb1 F | 5′-CCT AGT GCT GGG ATT ACA AG-3′ | Genotype |
| Arrb1 R | 5′-CAT AGC CTG AAG AAC GAG AT-3′ | Genotype |
| Arrb2 L WT | 5′-ACA GGG TCC ACT TTG TCC A -3′ | Genotype |
| Arrb2 mutant | 5′-GGG GGT GGG GAG GGG TGT TAG-3′ | Genotype |
| Arrb2 R WT | 5′-GCT AAA GCG CAT GCT CCA GA -3′ | Genotype |
| LysM-Cre Common | 5′-CTT GGG CTG CCA GAA TTT CTC-3′ | Genotype |
| LysM-Cre Mutant | 5′-CCC AGA AAT GCC AGA TTA CG-3′ | Genotype |
| LysM-Cre Wildtype | 5′-TTA CAG TCG GCC AGG CTG AC-3′ | Genotype |

**Supplementary Table 3. TranSignal™ SH3 Domain Array kit information.**

| CRK | Itk | SJHUA | Lyn1 | Yes1 | MLPK3 | Cortactin | SPCN | CCB4 | Amphiphysin |
| --- | --- | --- | --- | --- | --- | --- | --- | --- | --- |
| CRK | Itk | SJHUA | Lyn1 | Yes1 | MLPK3 | Cortactin | SPCN | CCB4 | Amphiphysin |
| NOF | VAV2 | Hck | FYB | Src | Nebulin | SLK | FGR | EMP55 | Dlg2 |
| NOF | VAV2 | Hck | FYB | Src | Nebulin | SLK | FGR | EMP55 | Dlg2 |
| Abl(2) | HS1 | Tim | PS095 | RasGAP | BTK | PEXD | Y124 | NCK (3) | VAV |
| Abl(2) | HS1 | Tim | PS095 | RasGAP | BTK | PEXD | Y124 | NCK (3) | VAV |
|  |  | GST | TXK | ITSN (1) | ITSN (2) | Riz | PLCγ | Abl(1) | BLK |
|  |  | GST | TXK | ITSN (1) | ITSN (2) | Riz | PLCγ | Abl(1) | BLK |

**Supplementary Table 4. Data collection and refinement statistics**

|  | Src(SH3+SH2)-54CT(PRRRP motif) |  |
| --- | --- | --- |
| **Data collection** |  |  |
| Space group | P3_1_ 21 |  |
| Cell dimensions |  |  |
| *a*, *b*, *c* (Å) | 95.80, 95.80, 123.38 |  |
| Resolution (Å) | 82.99 - 3.54 (3.66- 3.54) * |  |
| *R*_merge_ (%) | 0.16(153.6) |  |
| *CC* (1/2) (%) | 99.2(76.4) |  |
| *I* / σ*I* | 13.3(2.3) |  |
| Completeness (%) | 97.4(99.9) |  |
| Redundancy | 19.1(18.4) |  |
|  |  |  |
| **Refinement** |  |  |
| Resolution (Å) | 19.97 - 3.50 |  |
| No. reflections | 8063 |  |
| *R*_work_ / *R*_free_ | 0.268 / 0.295 |  |
| Ramachandran |  |  |
| Favored (%) | 95.36 |  |
| Allowed (%) | 5.64 |  |
| Outlier (%) | 0 |  |
| R.m.s. deviations |  |  |
| Bond lengths (Å) | 0.002 |  |
| Bond angles (°) | 0.463 |  |
| PDB ID | 7YQE |  |

*Values in parentheses are for the highest-resolution shell.
