# Supplementary material for: Kisspeptin-10 binding to Gpr54 in osteoclasts prevents bone loss by activating Dusp18-mediated dephosphorylation of Src
Source: Nat Commun. 2024 Feb 12;15:1300. doi: 10.1038/s41467-024-44852-9 (PMC10861593; doi:10.1038/s41467-024-44852-9)
Supplement: Supplementary file 2 — Reporting Summary [file 41467_2024_44852_MOESM2_ESM.pdf]

nature portfolio

Corresponding author(s): Zhenxi Li
Last updated by author(s): Dec 11, 2023

Reporting Summary

Nature Portfolio wishes to improve the reproducibility of the work that we publish. This form provides structure for consistency and transparency in reporting. For further information on Nature Portfolio policies, see our Editorial Policies and the Editorial Policy Checklist.

Statistics

For all statistical analyses, confirm that the following items are present in the figure legend, table legend, main text, or Methods section.

n/a	Confirmed	
☐	☒	The exact sample size (*n*) for each experimental group/condition, given as a discrete number and unit of measurement
☐	☒	A statement on whether measurements were taken from distinct samples or whether the same sample was measured repeatedly
☐	☒	The statistical test(s) used AND whether they are one- or two-sided<br>*Only common tests should be described solely by name; describe more complex techniques in the Methods section.*
☐	☒	A description of all covariates tested
☐	☒	A description of any assumptions or corrections, such as tests of normality and adjustment for multiple comparisons
☐	☒	A full description of the statistical parameters including central tendency (e.g. means) or other basic estimates (e.g. regression coefficient) AND variation (e.g. standard deviation) or associated estimates of uncertainty (e.g. confidence intervals)
☐	☒	For null hypothesis testing, the test statistic (e.g. *F*, *t*, *r*) with confidence intervals, effect sizes, degrees of freedom and *P* value noted<br>*Give P values as exact values whenever suitable.*
☒	☐	For Bayesian analysis, information on the choice of priors and Markov chain Monte Carlo settings
☒	☐	For hierarchical and complex designs, identification of the appropriate level for tests and full reporting of outcomes
☒	☐	Estimates of effect sizes (e.g. Cohen's *d*, Pearson's *r*), indicating how they were calculated

Our web collection on statistics for biologists contains articles on many of the points above.

Software and code

Policy information about availability of computer code

Data collection	The raw mass spectrometry data were searched against the mouse IPI databases (version 3.86, released on June 28, 2012) using the Proteome Discoverer software suite (Thermo Scientific, San Jose, USA) utilizing a label-free quantification feature. Protein structure refinement was carried out using Phenix and with manual adjustments with Coot.
Data analysis	We used the ImageJ 1.52a software to quantify the protein bands intensity, the Excel 2016 and GraphPad Prism 7 to do the graph figures and statistics, the NIS-Elements Viewer to quantify the degree of co-localization. BMD, and 3D models were analyzed using CTAn software (Bruker micro CT). 3D models were adjusted in CT Vox software (Bruker micro CT). Histomorphometric measurements were made using the OsteoMeasure Analysis System (Osteometrics, Atlanta)

For manuscripts utilizing custom algorithms or software that are central to the research but not yet described in published literature, software must be made available to editors and reviewers. We strongly encourage code deposition in a community repository (e.g. GitHub). See the Nature Portfolio guidelines for submitting code & software for further information.

Data

Policy information about availability of data

All manuscripts must include a data availability statement. This statement should provide the following information, where applicable:

- Accession codes, unique identifiers, or web links for publicly available datasets
- A description of any restrictions on data availability
- For clinical datasets or third party data, please ensure that the statement adheres to our policy

The data from mass-spectrometric analysis for identification of GPR554 binding proteins were deposited in PRoteomics IDEntifications Database (PRIDE) and the accession number is PXD038433 (https://www.ebi.ac.uk/pride/archive/projects/PXD038433). The other data supporting the findings of this study are available in the main figures, supplementary information and the source data file. Source data are provided with this paper.

Human research participants

Policy information about studies involving human research participants and Sex and Gender in Research.

Reporting on sex and gender	This study recruited 10 participants, with 4 males and 6 females.
Population characteristics	The participants in this study were diagnosed with giant cell tumor of bone on the spine.
Recruitment	The participants were recruited from Shanghai Changzheng Hospital
Ethics oversight	The Institutional Review Board and the research ethics committee of Shanghai Changzheng Hospital

Note that full information on the approval of the study protocol must also be provided in the manuscript.

Field-specific reporting

Please select the one below that is the best fit for your research. If you are not sure, read the appropriate sections before making your selection.

☒ Life sciences ☐ Behavioural & social sciences ☐ Ecological, evolutionary & environmental sciences

For a reference copy of the document with all sections, see nature.com/documents/nr-reporting-summary-flat.pdf

Life sciences study design

All studies must disclose on these points even when the disclosure is negative.

Sample size	No particular statistical methods were used to predetermine sample size. All sample sizes are shown in the figures legends or in the methods section of our manuscript, respectively. Sample size was determined according to our experience as well as literature reporting in terms of specific experiment.
Data exclusions	No data was excluded from the experiments.
Replication	All experimental findings were reliably reproduced. Multiple independent repeats were included for related experiments. Each experiment was performed for at least three times to make sure similar results are reproducible. Animal-related experiments experiments have been done once.
Randomization	For cell analysis, we plated cells in a random distribution and randomly assigned them to experimental groups. For in vivo animal analysis, all mice initially were randomized into different experimental groups.
Blinding	The investigators were not blinded to data collection and analysis for in vivo animal analysis, because we measured the value of bone parameters and gave different treatments among groups.

Reporting for specific materials, systems and methods

We require information from authors about some types of materials, experimental systems and methods used in many studies. Here, indicate whether each material, system or method listed is relevant to your study. If you are not sure if a list item applies to your research, read the appropriate section before selecting a response.

Materials & experimental systems

n/a	Involved in the study
	☒ Antibodies
	☒ Eukaryotic cell lines
☒	Palaeontology and archaeology
	☒ Animals and other organisms
☒	Clinical data
☒	Dual use research of concern

Methods

n/a	Involved in the study
☒	ChIP-seq
☒	Flow cytometry
☒	MRI-based neuroimaging

Antibodies

Antibodies used

p-Src (Tyr416), CST (#2101), WB (1: 1,000);
Src, CST (#2109), WB/IP/IF (1: 1,000/1:50/1:400);
Src, CST (#2110), WB/IF (1: 1,000/1:400);
Phospho-IKKα/β (Ser176/180), CST (#2697), WB (1: 1,000);
IKKβ, CST (#8943), WB (1: 1,000);
p-p38 (Thr180/Tyr182), CST (#9211), WB (1: 1,000);
p38, CST (#9212), WB (1: 1,000);
p- JNK(Thr183/Tyr185), CST (#9251), WB (1: 1,000);
JNK, CST (#9252), WB (1: 1,000);
p-Erk1/2(Thr202/Tyr204), CST (#4370), WB (1: 1,000);
Erk1/2, CST (#4695), WB (1: 1,000);
KISS1R, CST (#13776), WB/IP/IF/IHC (1:1,000/1:50/1:100/1:100);
KiSS-1, Santa Cruz (sc-101246), WB/ IHC (1: 500/1:50);
DUSP18, Santa Cruz (sc-376923), WB/IP/IF/IHC (1:500/1:20/1:100/1:100);
NFATc1, Santa Cruz (sc-7294), WB (1: 500);
Actin, CST (#3700), WB (1: 1,000);
VINCULIN, Sigma (V4505), WB (1: 5,000);
Rabbit IgG, CST (#2729), WB (1: 1,000);
GST-tag, CST (#2624), WB (1: 1,000);
Myc-tag, CST (#2272), WB (1: 1,000);
Rabbit HA-tag, CST (#3724), WB (1: 1,000);
Mouse HA-tag, Santa Cruz (sc-7392), WB (1: 1,000);
Mouse Flag-tag, Sigma (F3165), WB (1: 5,000);
Rabbit Flag-tag, Sigma (F7425), WB (1: 5,000);
peroxidase-conjugated mouse secondary antibody, Sigma (A4416), WB (1: 5,000);
peroxidase-conjugated rabbit secondary antibody, Sigma (A4914), WB (1: 5,000).

Validation

All these antibodies below are commercially available and all validated by the producers.
p-Src (Tyr416), CST (#2101), WB (1: 1,000);
https://www.cellsignal.cn/products/primary-antibodies/phospho-src-family-tyr416-antibody/2101?site-search
type=Products&N=4294956287&Ntt=2101&fromPage=plp&_requestid=4752896
Src, CST (#2109), WB/IP/IF (1: 1,000/1:50/1:400);
https://www.cellsignal.cn/products/primary-antibodies/src-36d10-rabbit-mab/2109?site-search-
type=Products&N=4294956287&Ntt=2109&fromPage=plp&_requestid=4753488
Src, CST (#2110), WB/IF (1: 1,000/1:400);
https://www.cellsignal.cn/products/primary-antibodies/src-l4a1-mouse-mab/2110?site-search-
type=Products&N=4294956287&Ntt=2110&fromPage=plp&_requestid=4753527
Phospho-IKKα/β (Ser176/180), CST (#2697), WB (1: 1,000);
https://www.cellsignal.cn/products/primary-antibodies/phospho-ikka-b-ser176-180-16a6-rabbit-mab/2697?site-search-
type=Products&N=4294956287&Ntt=2697&fromPage=plp&_requestid=4753577
IKKβ, CST (#8943), WB (1: 1,000);
https://www.cellsignal.cn/products/primary-antibodies/ikkb-d30c6-rabbit-mab/8943?site-search-
type=Products&N=4294956287&Ntt=8943&fromPage=plp&_requestid=4753685
p-p38 (Thr180/Tyr182), CST (#9211), WB (1: 1,000);
https://www.cellsignal.cn/products/primary-antibodies/phospho-p38-mapk-thr180-tyr182-antibody/9211?site-search-
type=Products&N=4294956287&Ntt=9211&fromPage=plp&_requestid=4753717
p38, CST (#9212), WB (1: 1,000);
https://www.cellsignal.cn/products/primary-antibodies/p38-mapk-antibody/9212?site-search-
type=Products&N=4294956287&Ntt=9212&fromPage=plp&_requestid=4753740
p- JNK(Thr183/Tyr185), CST (#9251), WB (1: 1,000);
https://www.cellsignal.cn/products/primary-antibodies/phospho-sapk-jnk-thr183-tyr185-antibody/9251?site-search-
type=Products&N=4294956287&Ntt=9251&fromPage=plp&_requestid=4753771
JNK, CST (#9252), WB (1: 1,000);
https://www.cellsignal.cn/products/primary-antibodies/sapk-jnk-antibody/9252?site-search-
type=Products&N=4294956287&Ntt=9252&fromPage=plp&_requestid=4753795
p-Erk1/2(Thr202/Tyr204), CST (#4370), WB (1: 1,000);
https://www.cellsignal.cn/products/primary-antibodies/phospho-p44-42-mapk-erk1-2-thr202-tyr204-d13-14-4e-xp-rabbit-
mab/4370?site-search-type=Products&N=4294956287&Ntt=4370&fromPage=plp
Erk1/2, CST (#4695), WB (1: 1,000);

https://www.cellsignal.cn/products/primary-antibodies/p44-42-mapk-erk1-2-137f5-rabbit-mab/4695?site-search-type=Products&N=4294956287&Ntt=4695&fromPage=plp
KISS1R, CST (#13776), WB/IP/IF/IHC (1:1,000/1:50/1:100/1:100);
https://www.cellsignal.cn/products/primary-antibodies/kiss1r-d9d7c-rabbit-mab/13776?site-search-type=Products&N=4294956287&Ntt=13776&fromPage=plp&_requestid=4754005
KiSS-1, Santa Cruz (sc-101246), WB/ IHC (1: 500/1:50);
https://www.scbt.com/p/kiss-1-antibody-24-q?requestFrom=search
DUSP18, Santa Cruz (sc-376923), WB/IP/IF/IHC (1:500/1:20/1:100/1:100);
https://www.scbt.com/p/dusp18-antibody-e-2?requestFrom=search
NFATc1, Santa Cruz (sc-7294), WB (1: 500);
https://www.scbt.com/p/nfatc1-antibody-7a6?requestFrom=search
Actin, CST (#3700), WB (1: 1,000);
https://www.cellsignal.cn/products/primary-antibodies/b-actin-8h10d10-mouse-mab/3700?site-search-type=Products&N=4294956287&Ntt=3700&fromPage=plp&_requestid=4754400
VINCULIN, Sigma (V4505), WB (1: 5,000);
https://www.sigmaaldrich.cn/CN/zh/product/sigma/v4505
Rabbit IgG, CST (#2729), WB (1: 1,000);
https://www.cellsignal.cn/products/primary-antibodies/normal-rabbit-igg/2729?site-search-type=Products&N=4294956287&Ntt=2729&fromPage=plp&_requestid=4754614
GST-tag, CST (#2624), WB (1: 1,000);
https://www.cellsignal.cn/products/primary-antibodies/gst-tag-26h1-mouse-mab/2624?site-search-type=Products&N=4294956287&Ntt=2624&fromPage=plp&_requestid=4754645
Myc-tag, CST (#2272), WB (1: 1,000);
https://www.cellsignal.cn/products/primary-antibodies/myc-tag-antibody/2272?site-search-type=Products&N=4294956287&Ntt=2272&fromPage=plp&_requestid=4754661
Rabbit HA-tag, CST (#3724), WB (1: 1,000);
https://www.cellsignal.cn/products/primary-antibodies/ha-tag-c29f4-rabbit-mab/3724?site-search-type=Products&N=4294956287&Ntt=3724&fromPage=plp&_requestid=4754690
Mouse HA-tag, Santa Cruz (sc-7392), WB (1: 1,000);
https://www.scbt.com/p/ha-probe-antibody-f-7?requestFrom=search
Mouse Flag-tag, Sigma (F3165), WB (1: 5,000);
https://www.sigmaaldrich.cn/CN/zh/product/sigma/f3165
Rabbit Flag-tag, Sigma (F7425), WB (1: 5,000);
https://www.sigmaaldrich.cn/CN/zh/product/sigma/f7425
peroxidase-conjugated mouse secondary antibody, Sigma (A4416), WB (1: 5,000);
https://www.sigmaaldrich.cn/CN/zh/product/sigma/a4416
peroxidase-conjugated rabbit secondary antibody, Sigma (A4914), WB (1: 5,000)
https://www.sigmaaldrich.cn/CN/zh/product/sigma/a4914.

Eukaryotic cell lines

Policy information about cell lines and Sex and Gender in Research

Cell line source(s)
RAW264.7, 293T, Sf9 cells were obtained from American Type Culture Collection. The Arrb1 and Arrb2 double knockout MEFs were kindly gifted from Dr. Robert J. Lefkowitz.

Authentication
All cell lines were routinely authenticated by analysis of cell growth rate and morphology.

Mycoplasma contamination
All cell lines were routinely tested to ensure the free of mycoplasma contamination.

Commonly misidentified lines (See ICLAC register)
None

Animals and other research organisms

Policy information about studies involving animals; ARRIVE guidelines recommended for reporting animal research, and Sex and Gender in Research

Laboratory animals
For laboratory animals, report species, strain and age OR state that the study did not involve laboratory animals.

Wild animals
No wild animals were used in this study.

Reporting on sex
Gpr54f/f mice, C57BL/6J, 4 months, 4 females and 3 males for bone mass and osteoclast activity assay.
Gpr54 cKO mice, C57BL/6J, 4 months, 4 females and 3 males for bone mass and osteoclast activity assay.
Kiss1 f/f mice, C57BL/6J, 4 months, 3 females and 3 males for bone mass and osteoclast activity assay.
Kiss1 cKO mice, C57BL/6J, 4 months, 3 females and 3 males for bone mass and osteoclast activity assay.
Gpr54f/f mice, C57BL/6J, 2 months, 6 females and 6 males for Elisa assay.
Gpr54 cKO mice, C57BL/6J, 2 months, 6 females and 6 males for Elisa assay.
Kiss1 f/f mice, C57BL/6J, 2 months, 6 females and 6 males for Elisa assay.
Kiss1 cKO mice, C57BL/6J, 4 months, 6 females and 6 males for Elisa assay.
Dusp18+/+ mice, C57BL/6J, 4 months, 3 females and 4 males for bone mass and osteoclast activity assay.
Dusp18-/- mice, C57BL/6J, 4 months, 2 females and 3 males for bone mass and osteoclast activity assay.

Dusp18-/- mice treated with KX2-391, C57BL/6J, 4 months, 2 females and 3 males for bone mass and osteoclast activity assay.
Dusp18-/- mice treated with (DSS)*6-KP-10, C57BL/6J, 4 months, 2 females and 3 males for bone mass and osteoclast activity assay.
Gpr54+/+ mice, C57BL/6J, 2 months, 3 females and 3 males for bone mass assay, 6 males and 6 females for Elisa assay.
Gpr54-/- mice, C57BL/6J, 2 months, 3 females and 3 males for bone mass assay, 6 males and 6 females for Elisa assay..
Kiss1 +/+ mice, C57BL/6J, 2 months, 3 females and 3 males for bone mass assay, 6 males and 6 females for Elisa assay..
Kiss1 -/- mice, C57BL/6J, 2 months, 3 females and 3 males for bone mass assay, 6 males and 6 females for Elisa assay..
Gpr54+/+ mice, C57BL/6J, 1, 2, 8, 8 (OVX), 24 weeks, 1 females and 4 males for TRAP staining of calvaria.
Gpr54-/- mice, C57BL/6J, 1, 2, 8, 8 (OVX), 24 weeks, 1 females and 4 males for TRAP staining of calvaria.
Kiss1 +/+ mice, C57BL/6J, 1, 2, 8, 8 (OVX),24 weeks, 2 females and 3 males for TRAP staining of calvaria.
Kiss1 -/- mice, C57BL/6J, 1, 2, 8, 8 (OVX),24 weeks, 2 females and 3 males for TRAP staining of calvaria.
Gpr54+/+ mice, C57BL/6J, 6 weeks, 3 females and 3 males for double calcein labeling and Goldner's Masson trichrome staining.
Gpr54-/- mice, C57BL/6J, 6 weeks, 3 females and 4 males for double calcein labeling and Goldner's Masson trichrome staining.
Kiss1 +/+ mice, C57BL/6J, 6 weeks, 3 females and 3 males for double calcein labeling and Goldner's Masson trichrome staining.
Kiss1 -/- mice, C57BL/6J, 6 weeks, 3 females and 3 males for double calcein labeling and Goldner's Masson trichrome staining.
Gpr54f/f mice, C57BL/6J, 6 weeks, 2 females and 2 males for double calcein labeling.
Gpr54 cko mice, C57BL/6J, 6 weeks, 2 females and 2 males for double calcein labeling.
Kiss1 f/f mice, C57BL/6J, 6 weeks, 2 females and 2 males for double calcein labeling.
Kiss1 cko mice, C57BL/6J, 6 weeks, 2 females and 2 males for double calcein labeling.
Dusp18 +/+ mice, C57BL/6J, 6 weeks, 3 females and 3 males for Goldner's Masson trichrome staining.
Dusp18 -/- mice, C57BL/6J, 6 weeks, 3 females and 3 males for Goldner's Masson trichrome staining.
Sham-operated mice, C57BL/6J, 4 months, 6 females treated with vehicle for bone mass and osteoclast activity assay (n=6), IHC staining (n=3).
Sham-operated mice, C57BL/6J, 4 months, 6 females treated with 50 nmol/kg (DSS)*6-Kp-10 for bone mass and osteoclast activity assay
ovariectomized mice, C57BL/6J, 4 months, 6 females treated with vehicle for bone mass and osteoclast activity assay (n=6), IHC staining (n=3).
ovariectomized mice, C57BL/6J, 4 months, 6 females treated with 50 nmol/kg (DSS)*6-Kp-10 for bone mass and osteoclast activity assay.
Sham-operated mice, C57BL/6J, 4 months, 4 females treated with vehicle for Von Kossa staining, 6 females treated with vehicle for Elisa assay.
ovariectomized mice, C57BL/6J, 4 months, 3 females treated with vehicle for Von Kossa staining, 6 females treated with vehicle for Elisa assay.
ovariectomized mice, C57BL/6J, 4 months, 6 females treated with 1 nmol/kg Kp-10 for Von Kossa staining.
ovariectomized mice, C57BL/6J, 4 months, 6 females treated with 10 nmol/kg Kp-10 for Von Kossa staining.
ovariectomized mice, C57BL/6J, 4 months, 5 females treated with 1nmol/kg (DSS)*6-Kp-10 for Von Kossa staining.
ovariectomized mice, C57BL/6J, 4 months, 6 females treated with 10 nmol/kg (DSS)*6-Kp-10 for Von Kossa staining and for Elisa assay.

Field-collected samples

This study did not involve any field-collected samples.

Ethics oversight

Animal study was approved by the Institutional Animal Care and Use Committee at East China Normal University m20140907

Note that full information on the approval of the study protocol must also be provided in the manuscript.
